# SOX9-positive pituitary stem cells differ according to their position in the gland and maintenance of their progeny depends on context

**DOI:** 10.1126/sciadv.adf6911

**Published:** 2023-10-04

**Authors:** Karine Rizzoti, Probir Chakravarty, Daniel Sheridan, Robin Lovell-Badge

**Affiliations:** ^1^ Laboratory of Stem Cell Biology and Developmental Genetics, The Francis Crick Institute, London NW1 1AT, UK.; ^2^ Bioinformatics core, The Francis Crick Institute, London NW1 1AT, UK.

## Abstract

Stem cell (SC) differentiation and maintenance of resultant progeny underlie cell turnover in many organs, but it is difficult to pinpoint the contribution of either process. In the pituitary, a central regulator of endocrine axes, adult SCs undergo activation after target organ ablation, providing a well-characterized paradigm to study an adaptative response in a multi-organ system. Here, we used single-cell technologies to characterize SC heterogeneity and mobilization together with lineage tracing. We show that SC differentiation occurs more frequently than thought previously. In adaptative conditions, differentiation increases and is more diverse than demonstrated by the lineage tracing experiments. Detailed examination of SC progeny suggests that maintenance of selected nascent cells underlies SC output, highlighting a trophic role for the microenvironment. Analyses of cell trajectories further predict pathways and potential regulators. Our model provides a valuable system to study the influence of evolving states on the mechanisms of SC mobilization.

## Teaser

Pituitary stem cells are diverse and differentiate more than thought but only selected progeny persists according to need.

## INTRODUCTION

The pituitary gland controls the three major endocrine axes, reproduction, stress, and metabolism, and is involved in homeostasis; all under control of the hypothalamus. Its function requires modulation of hormonal output over short and long terms, and this is regulated at several levels, including rates and patterns of hormone secretion, synthesis and storage, and changes in endocrine cell numbers. Different mechanisms are known to underlie endocrine cell emergence: division of endocrine cells, differentiation from resident stem cells (SCs), and transdifferentiation, although the relevance of the latter is unclear. Under normal and stable conditions, it is thought that division from endocrine cell types is sufficient for the typical low cell turnover seen in the pituitary (*1*). This has been directly shown in corticotrophs using a genetic trick where cell division induces apoptosis of proopiomelanocortin (POMC) expressing cells. A gradual loss of corticotrophs is observed in these pituitaries in support of the role of these endocrine cells in their own replacement (*2*). In agreement, long term lineage tracing of adult SOX2 and SOX9 positive (+ve) pituitary SCs in steady-state conditions suggests that SCs differentiate rarely and thus do not substantially participate in cell turnover (*3*, *4*). Furthermore, adult ablation of the SCs does not appear to affect pituitary function (*5*). In contrast, when the regenerative or adaptative potential of the gland is stimulated, either by endocrine cell ablation (*6*) or target organ ablation (*3*), respectively, SC-derived endocrine cells of the appropriate type emerge. Furthermore, hypertrophy of the targeted endocrine cell type is observed. In this latter paradigm, differentiation from SCs appears to be the main process underlying emergence of corticotrophs after adrenalectomy (Ax) (*2*), providing a well-characterized multi-organ system to explore mechanisms of SC activation.

Here, we explore the characteristics of pituitary SC fate acquisition using single-cell approaches combined with lineage tracing analyses. We observe that SOX9 +ve pituitary SCs comprise several functionally distinct sub-populations, located in the ventral and dorsal part of the epithelium lining the pituitary cleft and in the anterior pituitary parenchyma. We then explored mechanisms underlying SC mobilization after target organ ablation through characterizing cell trajectories and gene regulatory networks (GRNs) potentially involved in cell fate acquisition. We observed that while fate acquisition of the targeted cell type (corticotrophs after Ax and gonadotrophs after gonadectomy) was efficiently induced, differentiation toward all endocrine cell types unexpectedly also took place. Examination of longer-term outcomes using lineage tracing further showed that only a proportion of the differentiated progeny is retained, specifically that of the targeted cell type. More precisely, we only observe SC-derived corticotrophs after Ax, and lactotrophs in females, after selective estrogen receptor modulator (SERM) treatment. Therefore, while pituitary SC differentiation appears both more frequent and diverse than previously thought, our results suggest that the selective maintenance of nascent cells, according to perceived pathophysiological need and likely sex-dependent hormonal cues, is an important regulatory step controlling their adaptative response. Our model thus allows characterization of this response after pathophysiological manipulations that may well relate to normal life-changing events, such as puberty, pregnancy, and lactation.

## RESULTS

### Distinct regulatory pathways govern anterior lobe and intermediate lobe SC identity

**Fig. 1.**
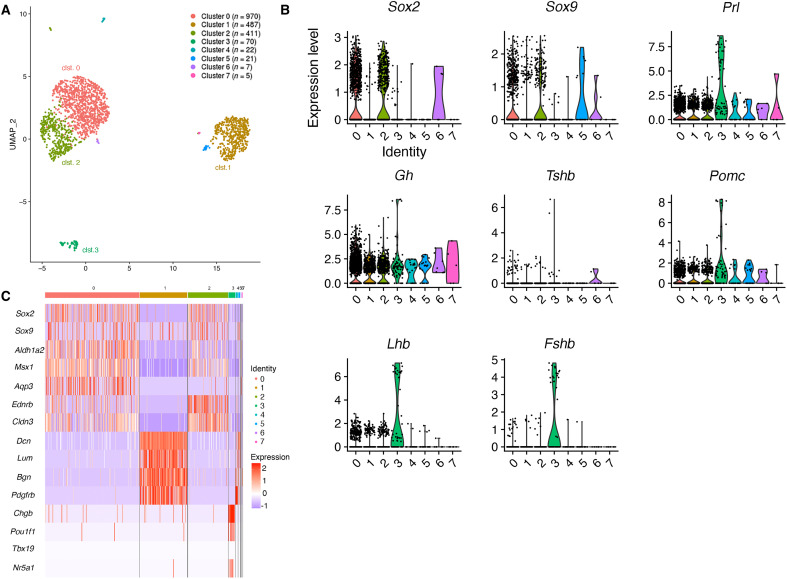
Single-cell analysis of the *Sox9^iresGFP/+^* population in male pituitaries. (**A**) UMAP clustering of *Sox9^iresGFP/+^* cells. (**B**) Expression levels of *Sox2* and *Sox9* were examined to identify SCs (*9*) while genes encoding for hormones (*Gh*, *Prl*, *Tsh*β, *Lhβ*, *Fshβ*, and *Pomc*) were examined to distinguish committed cells (*Pou1f1*) for somatotrophs, lactotrophs, and thyrotrophs. (**C**) Heatmap displaying selected markers for the main clusters.

The anterior lobes (ALs) of three *Sox9^iresGFP/+^* adult male pituitaries were dissociated and the transcriptome of single Green Fluorescent Protein (GFP) +ve cells were analyzed after fluorescence-activated cell sorting (FACS), as previously described (*3*). After quality checks, 1993 cells were retained for further analysis; clustering was performed by generating Uniform Manifold Approximation and Projection (UMAP) plots. Three major clusters were identified (Fig. 1A). Coexpression of *Sox2* and *Sox9* in clusters 0 and 2 (Fig. 1B and fig. S1), representing 69% of the cells, suggested that these were likely to represent SCs. The presence of two SC clusters had previously been reported in whole pituitary datasets (*7*, *8*). The remaining cluster (cluster 1), representing 24% of the dataset, comprised cells where *Sox*9 was detected, but where *Sox2* was greatly reduced or absent. Finally, cells each expressing a different hormone and the corresponding lineage specifying factors, were grouped within a single smaller cluster (cluster 3) representing 3% of the cells (Fig. 1B and fig. S1). SOX9 is not expressed in any endocrine cell in quiescent conditions (*9*). However, we observed some rare SOX9;Adrenocorticotropic hormone (ACTH) +ve cells, and more numerous GFP;ACTH +ve cells in *Sox9^iresGFP/+^* animals exclusively after activation of the SCs by pituitary target organ ablation (*3*) (fig. S2). This suggests that differentiating, and recently differentiated cells are present in the SOX9iresGFP fraction due to persistence of the GFP, which correlates with what has been suggested to happen in both human and mouse pituitaries, where hormone-positive cells were included in the SC compartment of whole pituitary single-cell RNAseq datasets (*10*). In addition, a small proportion of contaminating pituitary cells (estimated to represent 25% of cluster 3) is likely to be present (fig. S3). Therefore, most cells present in cluster 3 are likely to represent cells committed toward an endocrine fate, with the largest population [41% of cluster 3 cells expressing *Prolactin* (*Prl*)].

**Fig. 2.**
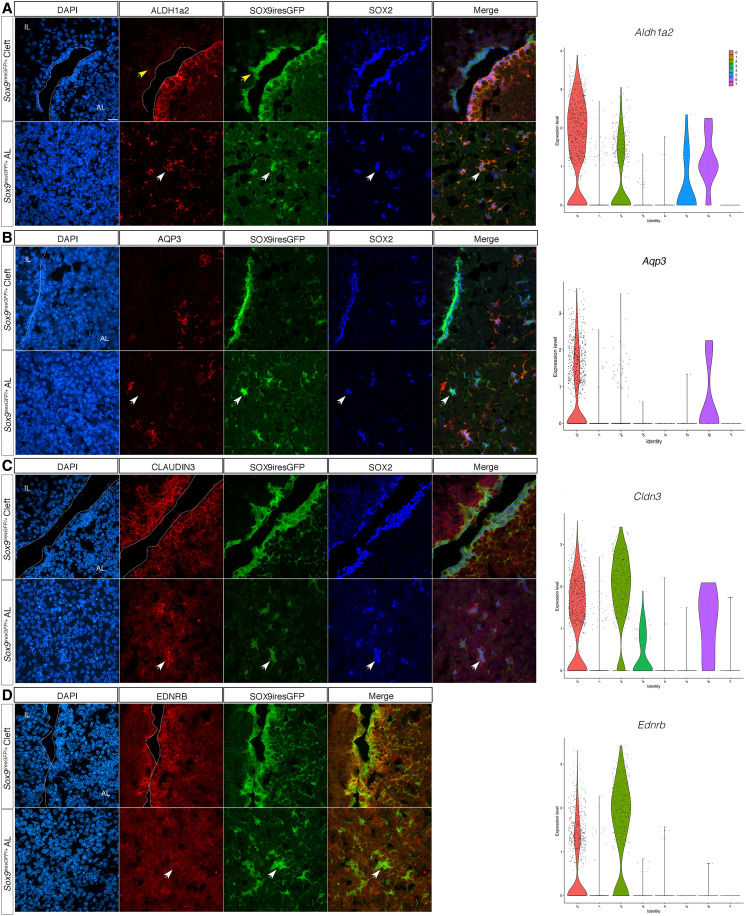
Differential expression of SC cluster markers. (**A**) Coimmunofluorescence for ALDH1A2, GFP, and SOX2 in a male SOX9iresGFP pituitary section. ALDH1A2 is exclusively expressed in SOX2;SOX9 AL SC (the yellow arrow shows its absence from IL SC, and the white arrow shows its expression in parenchymal SC). *Aldh1a2* violin plot shows enrichment in cluster 0. (**B**) Coimmunofluorescence for AQP3, GFP, and SOX2 in a male SOX9iresGFP pituitary section. AQP3 is expressed in 66% of SOX2 +ve parenchymal SC (white arrow shows SOX2;SOX9iresGFP parenchymal SC not expressing AQP3; counting was performed on *n* = 3 animals). *Aqp3* violin plot shows enrichment in cluster 0. (**C**) Coimmunofluorescence for CLAUDIN3, GFP, and SOX2 in a male SOX9iresGFP pituitary section. CLAUDIN3 is exclusively expressed in SOX2;SOX9 SC. *Cldn3* UMAP shows enrichment in cluster 2. (**D**) Coimmunofluorescence for EDNRB and GFP in a male SOX9iresGFP pituitary section. EDNRB is exclusively expressed in SOX9 cleft SC. *Ednrb* UMAP shows enrichment in cluster 2. Similar expression profiles were observed in females for all markers. The scale bar in (A) represents 10 μm for all panels. White arrows show absence of expression in parenchymal SC. AL, anterior lobe; IL intermediate lobe.

The two SC clusters were further explored. Analysis of differentially expressed genes (Fig. 1C and fig. S1B) shows that clusters 0 and 2 are relatively similar and are mostly distinguished by differences in levels of expression of specific genes. One notable exception is *Aqp3*, encoding for a water channel, which is specific for cluster 0. In contrast, *Aldh1a2*, encoding for an enzyme involved in retinoic acid (RA) synthesis, and the transcription factor *Msx1* were preferentially present in cluster 0, while *Ednrb*, encoding for the endothelin B receptor, and the cell adhesion molecule *Claudin3* were both expressed at higher levels in cluster 2. RA is required during pituitary morphogenesis (*11*, *12*) and *Msx1* interacts with SOX2 for *Prop1* regulation (*13*), while nothing is known about *Ednrb* or *Claudin3* in pituitary SCs. Conservation of most of these markers in SCs from whole pituitary datasets suggests that our two SC clusters correlate with those observed by others (*7*). In the gland, expression of the five markers is restricted to SCs (Fig. 2 and fig. S4). In the epithelium lining the cleft, cluster 0 markers ALDH1a2 and MSX1 are expressed exclusively on the AL side (Fig. 2 A and fig. S4B), while cluster 2 markers EDNRB and CLAUDIN-3 (Fig. 2, C and D) are present on both sides. In the parenchyma ALDH1a2, MSX1 and CLAUDIN-3 colocalize with SOX2/9 while expression of EDNRB is not detectable. In situ hybridization for *Ednrb* confirms a clear enrichment in cleft-lining cells (fig. S5). AQP3, whose transcript is present in about half of cluster 0 cells, is exclusively detected in the parenchymal SCs, revealing further heterogeneity between cleft-lining and parenchymal populations. In addition, it is only expressed in a subset of SOX2;SOX9 positive parenchymal SCs (Fig. 2B), demonstrating the existence of parenchymal subpopulations. Examination of the five markers in single-cell RNAseq datasets from whole pituitaries confirms that the strongest, and sometimes exclusive, expression is seen in SCs (*14*) (fig. S4). Altogether, these results suggest that cluster 0 is enriched in AL SCs, while cluster 2 has a more generic pituitary SC identity.

Pathway analysis in cluster 0 (table S1) highlighted activity of the NOTCH and WNT pathways and the presence of Ephrin signaling, as previously reported in pituitary SCs (*15*, *16*). Cluster 2 cells are characterized by expression of cytoskeleton remodeling and adhesion genes consistent with the epithelial to mesenchymal transition (EMT)-like process required during pituitary progenitor cell fate acquisition (*17*). In conclusion, regionalization of these two SC subpopulations is underlined by the expression of genes involved in the activity of specific regulatory pathways, such as RA signaling in AL SCs. Further investigation is required to understand what distinguishes AQP3-positive and -negative parenchymal SCs.

Folliculo-stellate (FS) cells are specialized AL glial cells (*18*). These express S100b in the rat, but not in the mouse [(*19*, *20*), fig. S6]. To clarify their relationship with SCs, we examined expression of *Aldolase C*, a marker for rodent FS cells (*20*), and of *Slc152a*, which encodes a transporter responsible for the FS-specific import of AMCA (*21*). Both are selectively present in SCs, in clusters 0 and 2 (fig. S6A). Furthermore, in a whole pituitary dataset, *Sox2* and *Aldoc* are exclusively present in the SC cluster (fig. 6B). Altogether, these results confirm, as suggested earlier (*9*, *22*–*24*), that in the mouse, FS cells and SCs are overlapping populations. In conclusion, single-cell analysis of the *Sox9^iresGFP/+^* pituitary fraction successfully resolved heterogeneity of this population, suggesting differential activity of signaling pathways, and clarified the relationship between SCs and FS cells.

### A population of parenchymal SOX9;PDGFRβ +ve cells is present in the pituitary

**Fig. 3.**
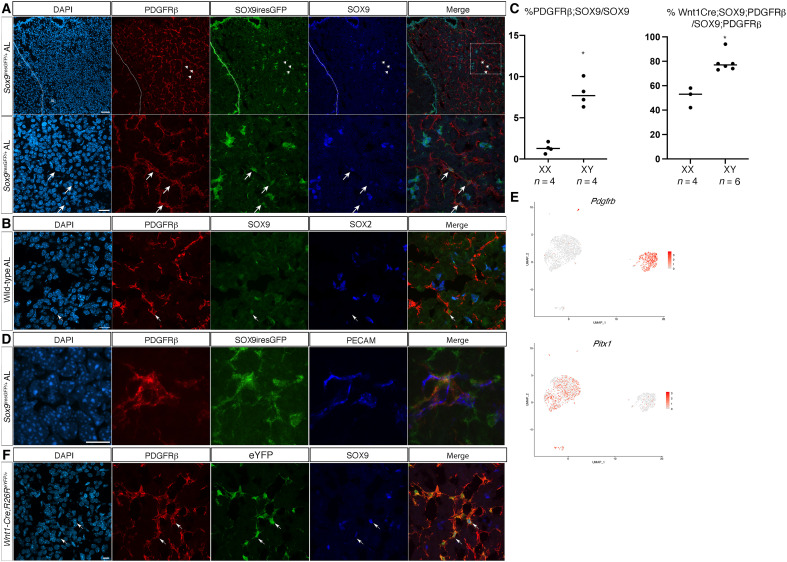
Characterization of a SOX9;PDGFRβ +ve AL cell population. (**A**) Coimmunofluorescence for PDGFRβ, GFP, and SOX9 in a SOX9iresGFP male AL section. Triple +ve cells are indicated (arrows). The bottom panels represent the magnification of the region indicated on the merge panel above. The dotted line indicates the pituitary cleft. (**B**) Coimmunofluorescence for PDGFRβ, GFP, and SOX2 in AL. A SOX9;PDGFRβ +ve cell that is negative for SOX2 (arrow). (**C**) Percentage of GFP;SOX9;PDGFRβ/GFP;SOX9 +ve cells in *Sox9iresGFP* male and female AL sections (average 500 SOX9 cells counted/animal, *P* = 0.029). In SOX9iresGFP pituitary sections, parenchymal SOX9;GFP were captured (table S4). Among these, the percentage of those also positive for PDGFRβ cells was determined. Percentage of SOX9;PDGFRβ +ve cells of neural crest origin (*P* = 0.024; Mann-Whitney test). In *Wnt1Cre;R26R^eYFP^* pituitary sections, SOX9;PDGFRb cells were captured. Among these, the percentage of those also positive for WNT1Cre;ReYFP cells was determined (table S4). (**D**) Coimmunofluorescence for PDGFRβ, GFP, and PECAM in a Sox9iresGFP AL section showing a SOX9;PDGFRβ +ve cell resembling a pericyte bridging PECAM +ve capillaries. (**E**) UMAP for *Pitx1* and *Pdgfrβ* showing that *Pdgfrβ* +ve cluster 1 cells do not express *Pitx1*. (**F**) Coimmunofluorescence for PDGFRβ, eYFP, and SOX9 in a *Wnt1-Cre;R26R^eYFP^* AL section. Triple +ve cells are shown (arrows). Scale bars, 50 μm and 20 μm (A, top and bottom panels, respectively) and 10 μm (D and F).

Cluster 1, representing 25% of the cells, is characterized by expression of PDGFRβ (Figs. 1C and 3E). In situ, PDGFRβ;SOX9 +ve cells are exclusively detected in the parenchyma. Expression of SOX9 in these double +ve cells is weaker than in SCs (Figs. 1B and 3A) and SOX2 is not detected (Figs. 1B and 3B), strongly suggesting that these cells are not pituitary SCs. In contrast with the in silico analysis, only 8 ± 1.62% SD of parenchymal SOX9 +ve cells express PDGFRβ in males (Fig. 3C). This discrepancy could be due to either an over-representation of cluster 1 cells, because epithelial SCs are more difficult to recover than parenchymal cells, or the difficulty in detecting the cells by immunofluorescence. We observe significantly fewer PDGFRβ;SOX9 +ve cells in females than in males (1.3 ± 0.6% SD, *P* = 0.029, Fig. 3C). PDGFRβ +ve pituitary cells are known to comprise a mural vascular network (*19*). While we did not attempt to fully characterize the SOX9;PDGFRβ population, we observe some SOX9;PDGFRβ cells in close contact with capillaries, displaying a pericyte-like morphology (Fig. 3D). In the embryo, vascular and mural cells have an extra-Rathke’s pouch origin, as they emerge from immigrating neural crest cells (*25*, *26*). Because cluster 1 cells do not express the generic pituitary marker *Pitx1* (Fig. 3E), these cells are not originating from the pouch. We thus analyzed a potential neural crest origin by performing WNT1-Cre lineage tracing; 79 ± 3.1% SEM of SOX9;PDGFRβ cells (*n* = 130 cells from four males) derive from WNT1-Cre +ve cells (Fig. 3, C and F), while in females, only 47.5 ± 5.5% SEM of the cells derive from the neural crest (*n* = 57 cells from three females, *P* = 0.024). Therefore, we propose that cluster 1 comprises a sexually dimorphic mural network with a dual embryonic origin. The pituitary capillary network, which controls endocrine output, adapts according to the physiological context and this is particularly important for gonadotroph function (*27*). The sex differences we observe may be related to this aspect.

### All endocrine cell types are present in the *Sox9^iresGFP/+^* +ve compartment after SC mobilization

**Fig. 4.**
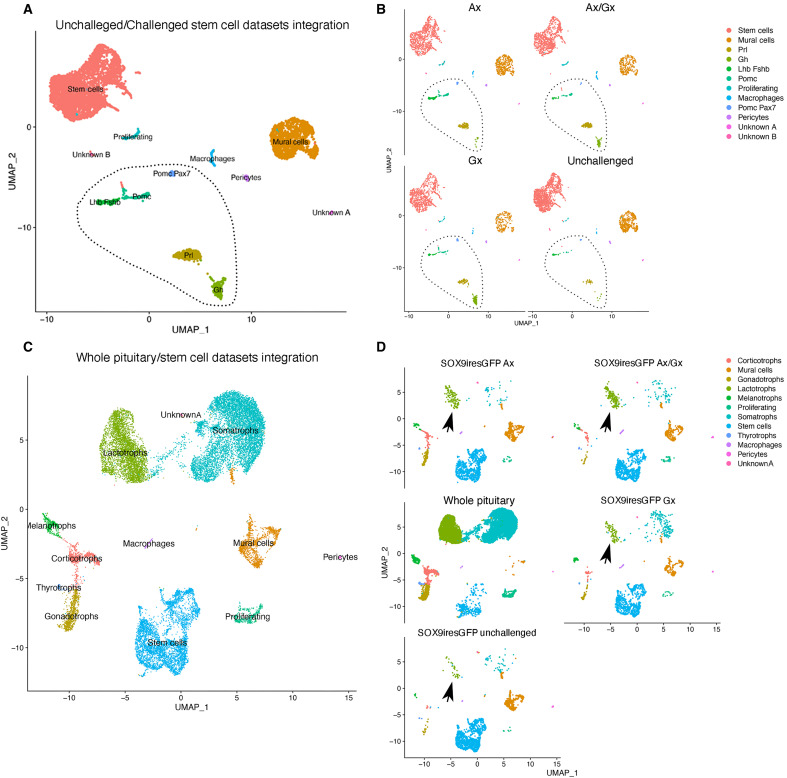
Target organ ablation induces differentiation toward multiple endocrine lineages. (**A**) UMAP clustering for integrated datasets from sorted *Sox9^iresGFP/+^* cells from unchallenged, adrenalectomized (1915 cells), gonadectomized (1546 cells), and both adrenalectomized and gonadectomized (1880 cells) animals, 4 days after surgery. (**B**) UMAP clustering split by experiment showing the contribution of cells originating from each dataset. Cells expressing all pituitary hormones are present (dotted lines) in all conditions. (**C**) UMAP clustering for integrated datasets from sorted *Sox9^iresGFP/+^* cells and a whole pituitary dataset (*14*). (**D**) UMAP clustering split by experiment show that hormone +ve cells present in the *Sox9^iresGFP/+^* cells cluster with endocrine cells (black arrow pointing to lactotrophs).

We then performed single-cell RNAseq on activated SCs. Target organ ablation induces a transient proliferative wave in the gland, mostly affecting SCs, which can then give rise to endocrine cells (*2*, *3*, *28*). We analyzed the transcriptome of *Sox9^iresGFP/+^* male cells 4 days after removing the adrenals, the testes, or both. We integrated these datasets with that obtained from unchallenged animals and obtained a similar clustering of SCs (Figs. 1 and 4, A and B, and fig. S7A). A pairwise comparison for proportion test limited to SC clusters demonstrates a significant increase in proliferative cells after target organ ablation (fig. S7B), as previously shown (*3*). We also observe the emergence of distinct hormone +ve clusters representing in total 25 to 30% of all cells (Fig. 4, A and B) compared to 3.5% in the unchallenged dataset, suggesting increased differentiation. Target organ ablation is associated with increased secretion, and sometimes cell numbers, of the endocrine population regulating the resected organ. However, regardless of the glands removed, all endocrine cell lineages emerge in the SOX9iresGFP +ve fraction. While we expected *Pomc* +ve cells to prominently feature after Ax, these were outnumbered by *Prl* and *Luteinizing and Follicle-Stimulating hormones* (*Lhb*;*Fshb*) +ve cells (fig. S7C). Similarly, gonadectomies were expected to trigger commitment toward the gonadotroph cell fate, but *Growth Hormone* (*GH*) +ve cells were mostly observed. To characterize these *Sox9^iresGFP/+^*;hormone +ve cells, we compared them with endocrine cells by integrating our datasets with one from whole pituitaries (*14*). *Sox9^iresGFP/+^*;hormone +ve cells cluster with endocrine cells (Fig. 4, C and D). Therefore, they represent differentiated cells, at least transcriptionally. In conclusion, target organ ablation induces differentiation of SCs, but there is an unexpected lack of specificity in the endocrine cell types produced, with all hormonal markers being up-regulated.

### Cell trajectories are inferred after Ax

**Fig. 5.**
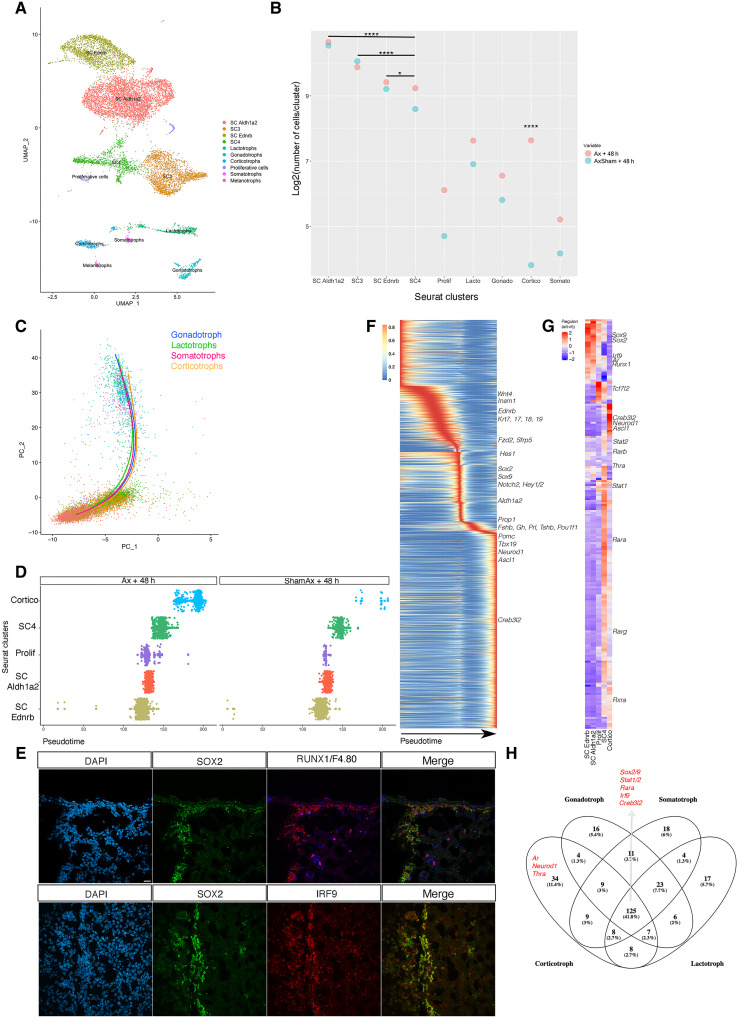
Trajectory analyses after Ax. (**A**) UMAP clustering for integrated datasets from sorted GFP +ve cells from unchallenged, adrenalectomized and sham + 48 h, and +4 days, all from *Sox9^iresGFP/+^* pituitaries. The data have been filtered to remove mural and vascular cells and outliers. Cluster color scheme is constant throughout the figure. (**B**) Pairwise comparison for proportion test performed on adrenalectomized and sham + 48 h. The proportion of corticotrophs after Ax is significantly different from all other cluster distributions. The proportion of cells in cluster SC4 is significantly different from all other SC cluster distributions (table S2). (**C**) Representation of endocrine cell trajectories on the PCA plot for integrated dataset. (**D**) Proportion of cells per cluster in the corticotroph trajectory comparing Ax and sham Ax. Differentiation is occurring predominantly after Ax. (**E**) Immunofluorescence on pituitary sections validates RUNX1 and IRF9 expression in SOX2 +ve SCs. RUNX1 is also expressed in F4.80 +ve macrophages. (**F**) Heatmap of 2245 genes associated with the corticotroph trajectory. (**G**) Heatmap of SCENIC analysis on the corticotroph trajectory. (**H**) Venn diagram of the common and unique regulons between the different endocrine trajectories (fig. S11).

We decided to focus on Ax because it represents the most efficient stimulus of SC differentiation after target organ ablation (*3*). We added an additional time point at day 2 after Ax and a sham control. These datasets were then integrated (Fig. 5A) with our initial dataset (Fig. 1) and the one performed 4 days after Ax (Fig. 4). UMAP clustering indicated that SCs were present in four distinct clusters (fig. S4). In addition to the *Ednrb* and *Aldh1a2* clusters, two SC clusters (SC3 and SC4) emerged (Fig. 5A). These were mostly but not exclusively populated by cells from the +48 h0 datasets (fig. S8). While more cells were sequenced in these +48 h00 datasets (up to three times more), we used a different chemistry (see Materials and Methods), which, despite Seurat batch correction tools, may explain detection of more genes and cells resulting in the emergence of these clusters. SC3 was characterized by higher expression of markers also present in the *Ednrb* and *Aldh1a2* clusters. SC4, mostly derived from the Ax+48 h00 dataset, showed coexpression of SC and endocrine markers, suggesting that it represents cells in a transitioning phase (fig. S9). Finally, endocrine cell clusters were detected (fig. S9), as observed previously (Fig. 4).

In sham-operated animals, 4.5% of hormone-expressing cells, mostly *Prl* +ve, were present, confirming our observations in unchallenged animals (table S2). Forty-eight hours after Ax, the proportion of hormone +ve cells had increased to 11%, and this had again doubled after 4 days. Because *Sox9iresGFP* expression stops as cells differentiate, the increasing proportion means that commitment toward differentiation augments during the 4 days after ablation. A pairwise proportion test between the 2 + 48 h0 datasets, sorted identically on the same day, showed that a shift occurs from SC4, while the proportion of cells from each dataset in the three SC clusters were comparable; from SC4, the proportion of cells from the Ax dataset becomes significantly increased compared to the control, fitting with its transitioning phase profile (Fig. 5B and fig. S9). At this shorter time point, 48 h00 after adrenal ablation, corticotrophs are the most significantly increased cell type, while 4 days after surgery, they were outnumbered by lactotrophs. This sharp and transient increase could represent the specific SC response to adrenal ablation.

We then performed trajectory analyses after principal components analysis (PCA) using Slingshot (*29*) using exclusively Ax+48 h and Sham Ax+48 h datasets. Trajectories finishing at each endocrine cell type were discovered, with a common root through SC clusters *Ednrb*, *Aldh1a2*, proliferative cells, and SC4, then ending at each endocrine cell type (Fig. 5C and figs. S10 and S11). In the corticotroph trajectory (Fig. 5D), the expression pattern of 2245 genes significantly correlates with pseudotime (Fig. 5F). The expression of known SC markers (*Ednrb*, *Sox2*, *Sox9*, and *Aldh1a2*) and signaling pathway members (*Notch2*, *Hes1*, *Hey1*, *Wnt4*, *Fzd2*, and *Sfrp5*) was gradually lost. Pathway analyses further suggest keratin filament remodeling, in agreement with epithelial-to-mesenchymal transition during differentiation. *Ismn1* (*30*) and *Prop1* (*17*), which are involved in cell fate acquisition, are also present during early phases. Conversely, the corticotroph specifier and marker genes *Neurod1*, *Ascl1*, *Tbx19*, *Pomc*, and *Creb3l2* (*31*) are up-regulated as cells differentiate. A sharp transition occurs as *Prop1* is up-regulated and is characterized by a transient up-regulation of *Pou1f1* lineage and gonadotroph (*Fshb*) genes. Comparisons with trajectories from other lineages reveal similar trajectories with a transient up-regulation of unrelated lineages markers (fig. S11). This could reflect permissive chromatin remodeling as endocrine cell fate acquisition occurs.

SCENIC analyses were then performed to explore gene regulatory networks (GRNs) (*32*) and compare them between the different endocrine lineages (Fig. 5, G and H, and fig. S11). As expected, *Sox2* and *9* regulons featured in SC clusters while *Ascl1*, *Neurod1*, and *Creb3l2* were present in corticotrophs. The thyroid (*Thra*) and the androgen (*Ar*) hormone receptor regulons are featured in corticotroph-differentiating SCs, suggesting that SCs can respond to peripheral signals. Pituitary SC GRNs were also discovered, such as those involving *Runx1* and *Irf9* whose expression was validated in SCs (Fig. 5, E, G, and H). RUNX1 (RUNt-related transcription factor 1) is known to control adult skin SC maintenance by regulating WNT signaling (*33*) and is shared between the corticotroph and gonadotroph trajectories. IRFs (interferon regulatory factors) are modulators of cell growth and differentiation beside their anti-viral functions. IRF9 associates with phosphorylated STAT1:STAT2 (signal transducer and activator of transcription) dimers, and all are present in our four endocrine SCENIC analyses (*34*). Finally, several RA factors were also present in all trajectories, supporting RA involvement during endocrine cell differentiation (Fig. 5H).

In conclusion, by adding +48 h00 datasets, we validated our previous observations, suggesting a low differentiation rate of SCs, mostly into lactotrophs in the intact and sham-operated animal. We refined the dynamics of the adaptative response after mobilization, showing a transient peak of corticotroph differentiation 48 hours after Ax. Finally, we were able to infer endocrine cell trajectories revealing common and exclusive factors during each endocrine cell fate acquisition.

### A proportion of SC progeny is selectively maintained

To examine predictions from our transcriptomic analyses, we performed two different types of lineage tracing experiment. First, we directly assessed expression of hormonal markers on plated SOX9 iresGFP FACSorted single cells by immunofluorescence in males and females, 4 days after Ax or sham surgery. Second, to follow mobilized SCs further, we used *Sox9^ires-CreERT2/+^; R26R^EYFP/+^* animals and examined progeny 10 to 11 days after Cre induction (a week after surgery because induction is mostly performed for 3 days before surgery).

**Fig. 6.**
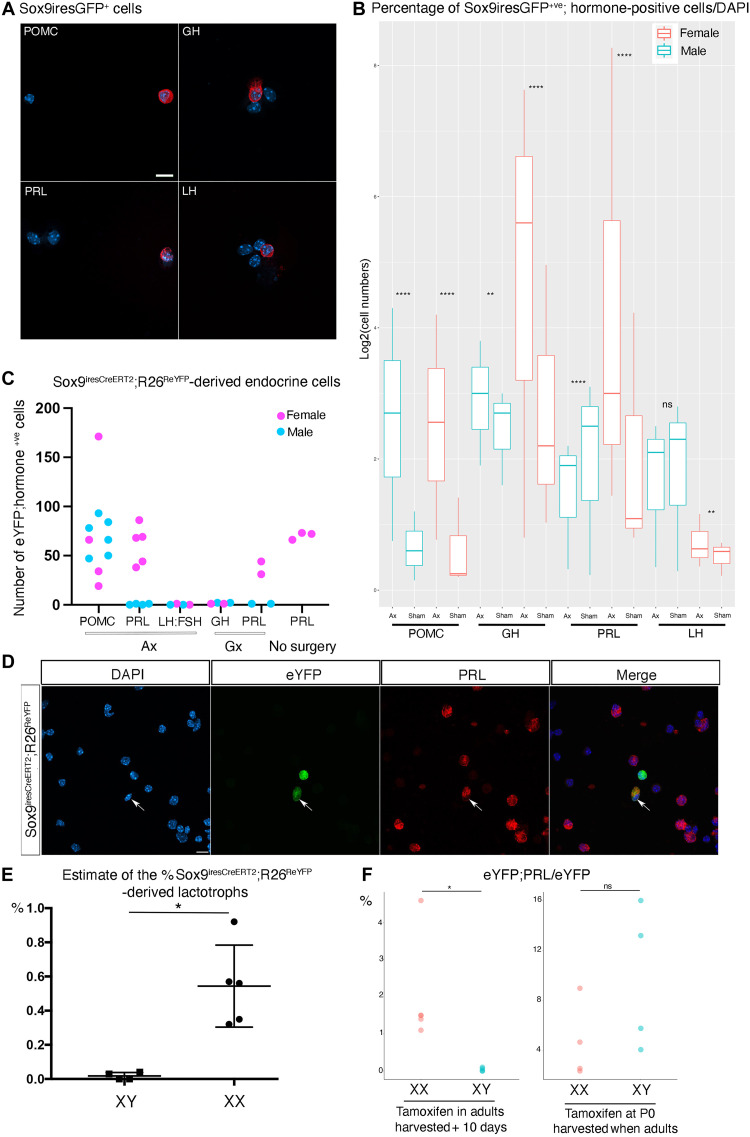
Analyses of single-cell RNAseq predictions by short- and long-term lineage tracing. (**A**) Immunofluorescence for hormonal markers on FACsorted female SOX9iresGFP +ve cells 4 days after Ax. (**B**) Percentages of hormone +ve/DAPI (4′,6-diamidino-2-phenylindole) from FACSorted SOX9iresGFP +ve cells. Counts were performed on plated pituitary cells harvested 4 days after surgeries. Pairwise for proportion tests were performed to assess significance of differences between Ax and Sham (table S3). (**C**) Numbers of hormone;eYFP +ve cells in *Sox9^iresCreERT2/+^;Rosa26^ReYFP^* pituitaries a week after surgeries or 10 days after tamoxifen treatment (no surgery), counted on comparable sections. Corticotrophs are observed in both sexes after Ax while lactotrophs are exclusively observed in females. LH;FSH cells were never observed after gonadectomies (*3*) and were thus not quantified. (**D**) Immunofluorescence for eYFP and PRL on dissociated nonsorted *Sox9^iresCreERT2/+^;Rosa26^ReYFP^* female pituitary cells. A PRL;eYFP +ve cell is indicated (arrow). (**E**) Estimation of the percentage of SC-derived lactotrophs. eYFP;PRL +ve cells were counted 10 days after tamoxifen treatment. Numbers were corrected for Cre induction efficiency, calculated in each animal as the percentage of cleft-lining SOX9;eYFP/SOX9 +ve cells (the cleft region was not dissociated). Because there is little proliferation in normal conditions in cleft-lining SCs, we assumed that the number of eYFP;SOX9 +ve cells reflected induction efficiency; 0.6% of lactotrophs originate from SCs 10 days after tamoxifen induction in females while we barely observed any in males (Mann-Whitney test performed on angular transformation of percentages, *P* = 0.0159). (**F**) Percentage of PRL;eYFP/eYFP +ve cells 10 days after tamoxifen treatment in 8- to 10-week-old animals, and in 8-week-old animals treated with tamoxifen as pups (eYFP;PRL/eYFP in *Sox9^iresCreERT2/+^;Rosa26^ReYFP^*). The proportion of SCs becoming lactotrophs is higher in adult females compared to males (*t* test, *P* = 0.03). This sex bias disappears when tamoxifen is administered in pups (*t* test test, *P* = 0.1825). Scale bars, 10 μm.

All hormone +ve cell types were found in the GFP +ve fraction in both experimental and control *Sox9^iresGFP/+^* animals, which matches our transcriptomic analyses (Fig. 6, A and B). After Ax, the number of POMC +ve cells increased the most, in agreement with our earliest dataset (Ax+48 h00). However, even if less efficiently stimulated after Ax, the proportion of most other endocrine cell types was comparable to that of corticotrophs. This again reflected our transcriptomic data. More precisely, we also observed a slight increase in somatotroph emergence in both sexes, while lactotrophs and, to a reduced extent, rare gonadotrophs appear to increase only in females.

We then carried out lineage tracing in *Sox9^ires-CreERT2/+^; R26R^EYFP/+^* animals (Fig. 6C). We examined the endocrine cell types predicted to be mostly induced by the single-cell analysis (fig. S3C) plus corticotrophs, because we knew these are present in the SC progeny after Ax (*3*). In contrast with our previous results (Fig. 6A), examination of pituitary sections exclusively revealed the presence of POMC;eYFP +ve corticotrophs after Ax, in comparable numbers in both sexes (Fig. 6C). In females, we additionally observed lactotrophs, and did so even without performing target organ ablation. Quantification of lactotroph emergence on dissociated pituitaries confirmed the sex bias (Fig. 6D). Correcting for CreERT2 induction efficiency, we estimate that, in females, SCs contribute to 0.6% of the total population of lactotrophs, and these SC-derived lactotrophs represent 2% of the eYFP +ve population (Fig. 5F).

The two genetic tools we used, *Sox9^iresGFP^* and *Sox9^ires-CreERT2/+^*, display inherent distinctions, such as the use of a high dose of the SERM tamoxifen, and purposes, where these may impair direct comparisons. However, while results from both transcriptomic analyses and immunofluorescence demonstrate that endocrine cells of all types are produced in comparable numbers in the short term, we clearly observe that corticotrophs are the only endocrine cells remaining in long-term lineage tracing experiments in males. This indicates a selective maintenance of nascent endocrine cells.

Alternatively, hormone-positive cells could acquire expression of SOX9 after surgeries, leading to the presence of SOX9;hormone +ve cells in the SOX9iresGFP fraction but not in the SOX9CreERT2 animals because tamoxifen was given before SC mobilization. There is no supporting evidence for this to happen, while we know that SCs differentiate (SOX9 +ve cells become hormone +ve). Furthermore, tamoxifen is known to persist in mice so we should still be able to see these cells in our lineage tracing experiments even if SOX9 becomes up-regulated after the surgeries (*35*). Finally, this discrepancy in the presence of hormone +ve cells in short-term versus long-term lineage tracing already exists in the intact animal because we see these cells in our unchallenged and sham single-cell RNAseq experiment, but ours and others’ lineage tracing analyses did not reveal any notable differentiation in normal conditions (*3*, *4*). Therefore, we think that the most likely explanation is the selective maintenance of a proportion of the SC progeny.

To further explore the sex bias in SC-derived lactotrophs, we administered tamoxifen to P0 *Sox9^ires-CreERT2/+^; R26R^EYFP/+^* pups and quantified differentiation into lactotrophs once they reached adulthood. There was no significant difference in the proportion of SC-derived lactotrophs between males and females (Fig. 5E), as previously observed (*3*). Therefore, male SC-derived lactotrophs can be produced, but this is temporally restricted. Furthermore, because the proportion of lactotrophs generated from male and female pups is similar, this suggests that tamoxifen, which can interfere with estrogen (E2) action, induces lactotroph emergence. If lactotroph emergence was a continuous process in females, their proportion would have been far superior to that observed in males. Our results thus reveal a temporal window of sensitivity in males, while female SCs remain responsive to the lactotroph promoting effect of tamoxifen until adulthood. It is unclear how E2 signaling is altered, and whether SC differentiation is induced or maintenance of nascent lactotrophs is permitted. Sexual dimorphism in SC activity has also been reported in another endocrine organ, the adrenals, where androgens regulate cell turnover (*36*).

### Hepatocyte growth factor trophic effect on pituispheres suggests that SCs and corticotrophs interact

**Fig. 7.**
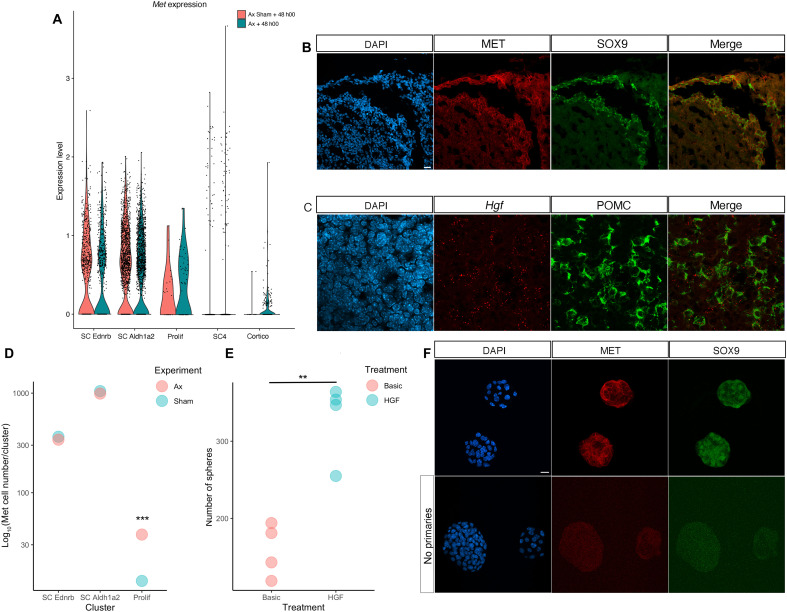
*Met*/MET and *Hgf* are expressed in SCs and corticotrophs, respectively, and the ligand has a trophic effect on pituispheres. (**A**) Expression levels of *Met* are shown in clusters belonging to the corticotroph trajectory (Fig. 5D). (**B**) Immunofluorescence for MET and SOX9 showing the receptor is expressed in SCs. (**C**) In situ hybridization (RNAscope) for *Hgf* and *Pomc* respectively showing that MET ligand is expressed in corticotrophs. *Hgf* is also predicted to be expressed in pericytes, which could explain signal outside corticotrophs. (**D**) More cells express *Met* after Ax specifically in the cluster comprising proliferating cells (chi-square test, *P* = 0.0005, table S3). (**E**) More pituispheres form when HGF (100 ng/ml) is added to basic medium (*t* test, *P* = 0.002, table S3, *n* = 4). (**F**) MET is expressed in SOX9 +ve pituispheres. Scale bar, 20 μm (B) and (C) and 10 μm (F).

The selective maintenance of specific nascent SC progeny, while the rest are lost, suggests that survival is dependent on local cues in their microenvironment. In the context of Ax, the corticotrophs are a likely source of signals. We thus re-examined our transcriptomic datasets, screening for signaling pathways potentially activated in SCs by corticotrophs. We found that the receptor tyrosine kinase *Met* is selectively up-regulated in SCs while its ligand *Hgf* is present in both corticotrophs and pericytes [Fig. 7, A to C, and fig. S12; (*37*)], suggesting interactions between these cell types. Furthermore, after Ax, the number of SCs expressing *Met* is increased, exclusively in those proliferating (Fig. 7D). This suggests a potential trophic effect of hepatocyte growth factor (HGF) on SCs that we explored in pituisphere assays (Fig. 7, E and F). Upon HGF treatment, the number of pituispheres is on average doubled compared to the control, demonstrating a trophic effect on SCs. Because the number of corticotrophs is increased after Ax and levels of HGF may thus potentially increase too, this pathway may mediate or at least participate in the increased SC proliferation observed after Ax. In agreement with a trophic role of the pathway in the pituitary, the receptor and its ligand are both widely expressed in human pituitary adenomas (*38*).

## DISCUSSION

While adult pituitary SCs appear superfluous for cell turnover in the unchallenged animal, their differentiation potential can be stimulated, for example, when the function of certain endocrine axes is compromised, such as when adrenals or gonads are ablated (*2*, *3*, *5*). Here, we have characterized SC heterogeneity and the modalities of endocrine cell emergence, by examining Sox9iresGFP single cells. We observe that a small percentage of pituitary SCs differentiate in the unchallenged animal, which correlates with single-cell RNAseq analyses performed on both human and mouse pituitaries where the presence of differentiating cells was also detected in the SC compartment (*10*). This proportion increases when SCs are mobilized. The paradigm we applied for SC mobilization, target organ ablation, is known to trigger a specific response in the pituitary: Adrenal ablation results in increased ACTH while gonadectomy is associated with increased gonadotropins. However, although there was a notable induction of corticotroph cell fate acquisition shortly after ablation, we unexpectedly observed increased differentiation toward all AL endocrine cell types. Nevertheless, in the longer term, lineage tracing experiments show that it is exclusively the targeted endocrine cell type that is maintained in the SC progeny. Therefore, after differentiation, selective maintenance of specific nascent cells is likely to be an important process regulating pituitary SC endocrine cell output, and it seems likely that this is controlled by the SC microenvironment. Increased sensitivity of nascent pituitary cells to apoptosis had been suggested before (*28*) and shown to increase after target organ ablation, in agreement with our results. Selective fitness of surviving cells in the embryo relies on access to particular trophic factors (*39*). In our Ax paradigm, the pituitary targets of the ablation, the corticotrophs, represent a likely source of signals, a notion re-enforced by the trophic effect of HGF we observe on pituispheres. Similarly, E2 is known to promote lactotroph proliferation and their emergence in embryos (*40*). We previously examined the effects of E2 on adult pituitary SC differentiation, but the prior administration of tamoxifen in our lineage tracing system may explain why we did not obtain meaningful results ((*3*). Here, our data suggest that tamoxifen treatment induces emergence of lactotrophs, both in male and female neonates, but exclusively in adult females. This in turn implies that this process is sensitive to E2, and that male development suppresses it. The presence of lactotrophs in the male SOX9iresGFP fraction, but not after lineage tracing, suggests that their maintenance is promoted in females. However, to further investigate these aspects, a tamoxifen-independent lineage tracing tool is required. In parallel with the likely role of corticotrophs on SCs after Ax, lactotrophs may be involved in the selective maintenance of nascent PRL cells emerging after tamoxifen treatment, because they are sensitive to its effect, given that prolactin secretion is increased by the SERM (*41*). In addition to the reliance on trophic factors, it is likely that nascent cell maintenance involves integration into homotypic endocrine networks (*42*). This is reminiscent of what is known for nascent neurons that are often produced in excess and whose persistence relies on synaptic connections (*43*). Although focusing on SCs enabled us to uncover regulators and unexpected modalities of the adaptative response, our analyses point toward an important role for the microenvironment and, in our multi-organ system, peripheric factors that are predicted to modulate the SC response. The thyroid (*Thra*) and the androgen (*Ar*) hormone receptors appear active in differentiating SCs, suggesting that they can respond to peripheral signals and may integrate this information to regulate pituitary plasticity, as recently proposed (*44*). Further investigations of both local and peripheral interactions are now required to better understand how this adaptative response is regulated in detail.

Differential clustering of SOX9iresGFP +ve SCs, trajectory analyses, and in situ validation of new markers revealed the activity of region-specific regulatory pathways. In agreement with what had been shown by others (*7*, *8*), we found two main populations. Only one of these two studies (*7*) shows a clustering of SCs based on the markers we characterized. However, detection of the expression of one marker exclusively in cleft SCs in the other study (*8*) suggests a clustering similarly based on SC localization. Because these studies were conducted on whole pituitaries, clustering is likely to have a different outcome from ours, where the dataset is restricted to SOX9iresGFP-positive cells. We can hypothesize that re-clustering of SCs extracted from these whole pituitary datasets should give rise to a similar resolution and markers. We further show here that these two populations correlate with differential localization, AL (ALDH1a2, MSX1 positive) versus more generic AL/intermediate lobe (IL) SC (CLDN3 positive) identity. This suggests that AL and IL SCs are regulated differently. We speculate that RA may be involved in AL SC differentiation, as shown in the embryo (*11*). The down-regulation of ALDH1a2 expression in IL SCs, which do not differentiate (*2*, *45*), provides an additional support to this hypothesis. The enrichment of EDNRb in the cells lining the cleft reveals a further level of heterogeneity between cleft lining and parenchymal cells. In the trajectory analyses, the upstream expression pattern of *Ednrb* implies that the SCs lining the cleft are in a more immature state than those in the parenchyma, which fits with this epithelium constituting the primary pituitary SC niche. Among the three endothelin ligands, *Edn3* is the most abundantly expressed in the gland, more precisely in SCs and pericytes (*37*). This suggests that this pathway could function in an autocrine or paracrine way in cleft SCs, because these are not in close contact with capillaries (*42*), which would make an interaction with the latter unlikely. EDNRB signaling has been shown to regulate melanocyte SC regeneration (*46*) and we are currently investigating whether this role is conserved in pituitary SCs. We have furthermore found that a subpopulation of parenchymal SCs is defined by AQP3 expression. It is unclear whether this correlates with the existence of functionally different subpopulations, such as supporting FS cells versus SCs, for example, or whether this reflects distinct SC states, but we are now able to identify these cells and investigate their role. Finally, the presence of RUNX, IFR, and STAT regulons during cell differentiation is also of potential interest because of the roles of these factors in different SC contexts, and because they have been shown to interact (RUNX/STAT and IFR/STAT). STAT transcription factors are activated by different cytokines and growth factors, in particular interleukin-6 whose role in the regenerative response of pituitary SCs was recently uncovered (*8*).

Our differentiation trajectories suggest the presence of a common root for all endocrine cell types, which only segregate as the terminal differentiation markers are up-regulated in the nascent cells. This process is likely to be fast because cells still contain GFP expressed from *Sox9iresGFP*. It is tempting to draw a parallel with embryonic pituitary progenitors, because these were similarly shown to all commit at the same stages (*47*). It was proposed that intrinsic factors and cell communication within the developing pituitary underlie specific endocrine cell fate acquisition, which, in a different context and with different modalities and outcomes, is what we hypothesize is happening in our model.

In conclusion, evaluation of single-cell transcriptomic predictions with short and long-term lineage tracing experiments has proved a robust strategy revealing aspects of pituitary SC adaptative responses. In circumstances where an exceptional pathophysiological need is perceived, nascent cells of the appropriate type are maintained. Understanding how the nascent cells can survive will help us to understand how SC input is controlled in the pituitary, but this could also be relevant in other tissues where SC activity appears largely superfluous. Finally, it is notoriously difficult to obtain mature cell types in vitro for use in regenerative medicine. Therefore, identifying trophic factors for nascent cells, which may be all that can be obtained for transplantation, appears necessary.

## MATERIALS AND METHODS

### Mice

All animal experiments carried out were approved under the UK Animals (Scientific Procedures) Act 1986 and under the project licenses n. 80/2405 and PP8826065. *Sox9^iresGFP/+^* (*Sox9^tm1Haak^*) (*48*), *Sox9^iresCreERT2/+^* (*Sox9^tm1(cre/ERT2)Haak^*) (*48*), *Wnt1Cre* [*(no gene)^tg(Wnt1-GAL4)11Rth^*] (*49*), and Rosa26^ReYFP/ReYFP^ [*Gt(ROSA)26Sortm1(EYFP)Cos*] (*50*) mice were maintained on a C57Bl6 background.

For CreERT2 induction in *Sox9^iresCreERT2/+^*;Rosa26^ReYFP/+^ animals, tamoxifen was administered for three consecutive days at a concentration of 0.2 mg/g body weight. For *Sox9^iresCreERT2/+^*;Rosa26^ReYFP/+^ pups induction, a single subcutaneous injection of tamoxifen (0.25 mg/g body weight) was performed. Target organ ablations were performed as previously described (*3*).

### Pituitary SC selection

The AL of freshly dissected 8- to 12-week-old pituitaries was minced and incubated for 15 min at 37°C in papain (1 mg/ml; Sigma-Aldrich, 10108014001), DNase I (10 μg/ml; Sigma-Aldrich, 10104159001), and Rock inhibitor (10 μg/ml; Abmole Bioscience M1817) in Hanks’ balanced salt solution (HBSS; Sigma-Aldrich, H9394). pH was adjusted by the addition of NaOH. The enzymatic solution was removed, and the cells were mechanically resuspended in HBSS supplemented with DNase I and Rock inhibitor, as above. Cells were FACsorted for GFP using a large nozzle (100 μm) and low pressure (20 psi) for optimal cell survival. For immunofluorescence, sorted cells were plated on Superfrost slides for 90 min in a cell culture incubator, fixed 20 min on ice in 4% paraformaldehyde (PFA), and immediately processed.

### Pituisphere culture

Dissociated ALs (see above) of male and female 7- to 12-week-old mice were seeded at 50 × 10^3^ cells/ml in pituisphere medium containing EGF and FGF (*9*). HGF (100 ng/ml; 2207-HG-025/CF, R&D Systems) was added to this basic medium in treated cultures. Medium was replaced every other day and spheres were manually and blindly counted after 7 days. Four independent repeats were performed.

### Single-cell sequencing

The concentration and viability of the single-cell suspension was measured using Trypan Blue and the Eve automatic cell counter (unchallenged and target organ ablation d4 samples) or acridine orange and propidium iodide with the Luna-FX7 automatic cell counter (Ax and Sham control d2 samples). Up to 10,000 cells were loaded on Chromium Chip and partitioned in nanoliter-scale droplets using the Chromium Controller and Chromium 10x 3 prime v2.0, Chromium Single Cell 3′ Reagent Kits User Guide (v2 Chemistry) (unchallenged and target organ ablation d4 samples) or Next GEM Single Cell reagents (3 prime v3.1) (Ax and Sham control d2 samples). Within each droplet, the cells were lysed, and the RNA was reverse-transcribed. All of the resulting cDNA within a droplet shared the same cell barcode. Illumina compatible libraries were generated from the cDNA using Chromium Next GEM Single Cell library reagents in accordance with the manufacturer instructions (10x Genomics). Final libraries are quality-checked using the Agilent TapeStation and sequenced using the Illumina HiSeq4000 or HiSeq2500, read configuration 26-8-0-98 (unchallenged and target organ ablation d4 samples) or NovaSeq 6000, read configuration 28–10–10-90. Where expression of specific genes in the whole pituitary gland is referenced as (*37*), we used this published dataset on the Loupe browser to examine expression of relevant markers.

### Bioinformatic analyses

Alignment, Seurat analysis, integration, and cluster annotation were performed using CellRanger and Seurat. Raw reads were initially processed by the Cell Ranger v.3.0.2 pipeline (*51*), which deconvolved reads to their cell of origin using the UMI tags, aligned these to the mm10 transcriptome (to which we added the eGFP sequence https://addgene.org/browse/sequence/305137/ to detect eGFP expressing cells) using STAR (v.2.5.1b) (*52*), and reported cell-specific gene expression count estimates. All subsequent analyses were performed in R v.3.6.0 (*53*) using the Seurat (v3) package (*54*). Genes were considered to be “expressed’ if the estimated (log10) count was at least 0.1. Primary filtering was then performed by removing from consideration cells expressing fewer than 50 genes and cells for which mitochondrial genes made up greater than 3 SD from the mean of mitochondrial expressed genes. PCA decomposition was performed and, after consideration of the eigenvalue “elbow-plots,” the first 20 components were used to construct the UMAP plots per sample. Multiple samples, generated in this study and published (*14*), were integrated using 2000 variable genes and Seurat’s CCA method. Cluster-specific gene markers were identified using a Wilcoxon rank sum test and the top 20 genes ranked by logFC per cluster were used to generate a heatmap. Clusters were annotated using label transfer methods within Seurat using the GSE120410 dataset (*14*). Clusters were further annotated using cell-specific signatures (table S5).

Cell trajectories were identified using the package “Slingshot” (version 1.4.0) (*29*), using the undifferentiated cluster as a starting point and the PCA coordinates. Lineages were identified showing specific trajectories ending in specific differentiated cells. Differential expression of genes between samples was further investigated for the corticotroph lineage with a correlation metric against pseudotime, using the “gam” package (version 1.20) (*55*). Heatmaps were made using the smoothed expression of differential genes, and the cells are ranked by increasing pseudotime.

To investigate transcription factor activity along pseudotime, the package SCENIC (*32*) was used. A list of 948 putative mouse regulatory binding sites found in promoter regions of expressed genes was used to identify shared regulatory networks. Regulon activity score per transcription factor was calculated using the AUCell function, where the enrichment of target genes was measured using the area under the curve of the target gene relative to the expression-based ranking of all genes. The top 200 regulons ranked by activity score were used to generate a binarized heatmap, where the cells were ranked by pseudotime.

### Immunofluorescence and RNAscope

Pituitaries were fixed after dissection by immersion in 4% PFA at 4°C. Immunofluorescent stainings were performed on cryosections as previously described (*56*). The following primary antibodies were used: Goat anti-SOX2 (Biotechne, AF2018), rabbit anti-SOX9 (a gift from F. Poulat, IGH Montpellier, France), rat anti-GFP (Nacalai Tesque, 04404-84), chick anti-GFP (Invitrogen, A10262), rabbit anti-EDNRb (Abcam, 117529), goat anti-MSX1 (R&D Systems, AF5045), rabbit anti-ALDH1A2 (Abcam, 75674, after antigen retrieval), rabbit anti-Claudin3 (Abcam, ab15102), rat anti-PECAM (BD Pharmigen, 550274), rat anti-PDGFRβ (eBiosciences, 14-1402-81), rabbit anti-AQP3 (Alomone Labs, AQP-003, after antigen retrieval), rabbit anti-Runx1 (Sigma-Aldrich, HPA004176), rabbit anti-IRF9 (Cell Signalling Technology, D9I5H), goat anti-Met (R&D Systems, AF 527), and hormone antibodies anti-LH, GH, ACTH, and PRL from the National Hormone Peptide Program (A.F. Parlow, Torrance, USA). Slides were then incubated with Alexa Fluor secondary antibodies. Imaging of stained tissue sections was performed on a Leica SPE microscope while imaging of plated cells was done on an Olympus spinning disk microscope.

RNAscope was performed on cryosections following the manufacturer’s instructions using *Hgf* (#456511), *Ednrb* (#473801), and *Pomc* (#314081) probes.

### Cell counts

Counts were performed blindly. For dissociated cell counts, slides stained by immunofluorescence were scanned; automated counts were then performed using QuPath-0.3.2 (https://qupath.github.io/). For *Sox9^iresCreERT2/+^*;Rosa26^ReYFP/+^;hormone counting on sections, double +ve cells were manually identified and each was imaged on a slide representing 1/5 of a pituitary. Images were then reviewed, and double +ve cells were counted. All counting results and tests are presented (tables S3 and S4).

### Statistics

Comparisons of the percentages of SOX9;PDGFRβ (Fig. 3) were performed after angular transformation using Mann-Whitney test on Prism. Pairwise for proportion test (fig. S4B, Figs. 5B and 6B, and table S3), *t* test (Figs. 6E and 7D), and chi-square test (Fig. 7) were performed using the Stats R package.
