# Supplementary material for: SOX9-positive pituitary stem cells differ according to their position in the gland and maintenance of their progeny depends on context
Source: Sci Adv. 2023 Oct 4;9(40):eadf6911. doi: 10.1126/sciadv.adf6911 (PMC10550238; doi:10.1126/sciadv.adf6911)
Supplement: Supplementary file 1 — Figs. S1 to S12 Tables S1 to S5 [file sciadv.adf6911_sm.pdf]

Science Advances
AAAS

Supplementary Materials for

SOX9-positive pituitary stem cells differ according to their position in the gland and maintenance of their progeny depends on context

Karine Rizzoti *et al.*

Corresponding author: Karine Rizzoti, karine.rizzoti@crick.ac.uk; Robin Lovell-Badge, robin.lovell-badge@crick.ac.uk

Sci. Adv. **9**, eadf6911 (2023)
DOI: 10.1126/sciadv.adf6911

This PDF file includes:

Figs. S1 to S12
Tables S1 to S5

Sup.Fig.1

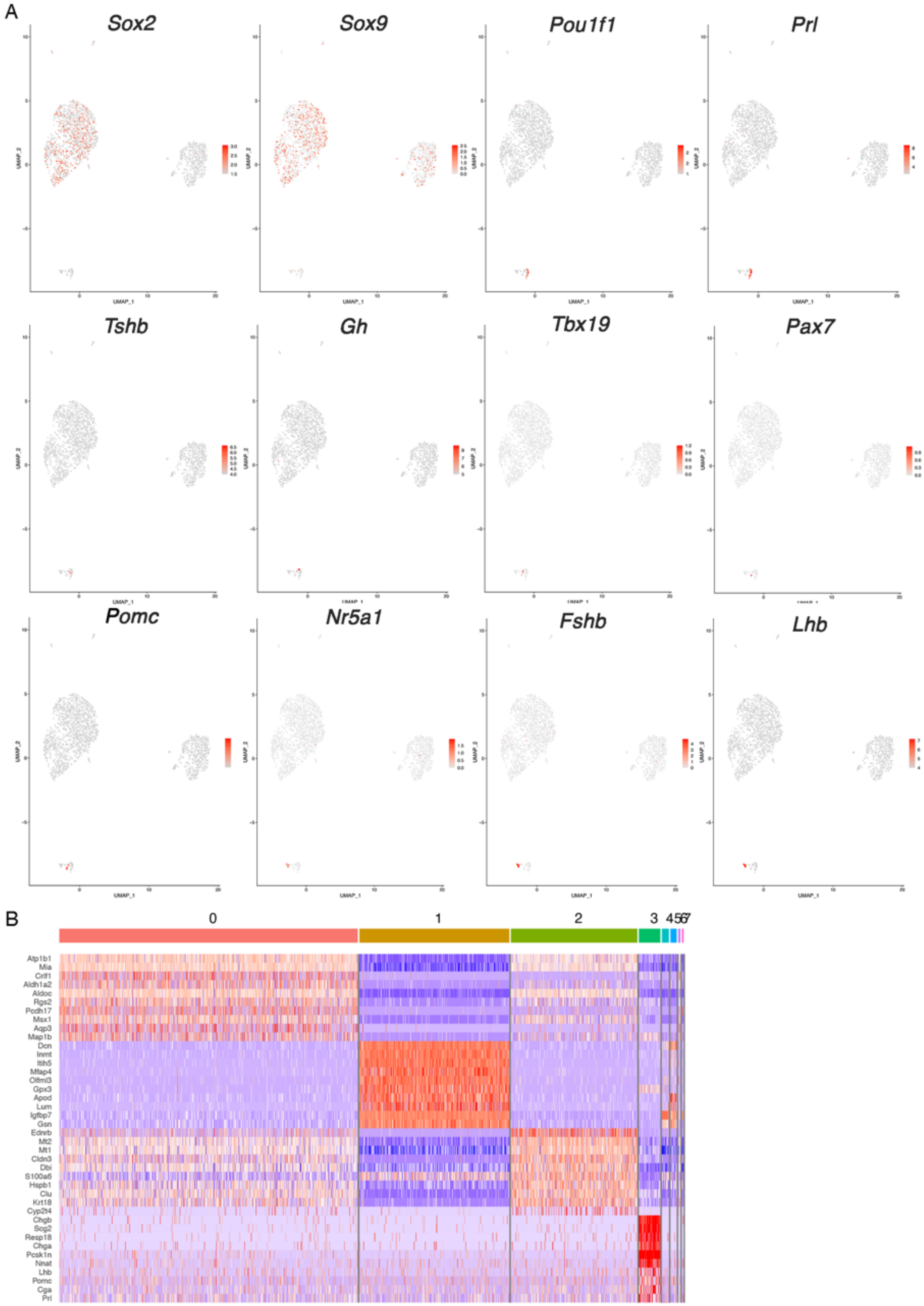
A
Sox2
Sox9
Pou1f1
Prl
Tshb
Gh
Tbx19
Pax7
Pomc
Nr5a1
Fshb
Lhb
UMAP_1
UMAP_2
B
0
1
2
3
4
5
6
7
Atp1b1
Mia
Crlf1
Aldh1a2
Aldoc
Rgs2
Pcdh17
Msx1
Aqp3
Map1b
Dcn
Inmt
Itih5
Mfap4
Olfml3
Gpx3
Apod
Lum
Igfbp7
Gsn
Ednrb
Mt2
Mt1
Cldn3
Dbi
S100a6
Hspb1
Clu
Krt18
Cyp2t4
Chgb
Scg2
Resp18
Chga
Pcsk1n
Nnat
Lhb
Pomc
Cga
Prl

Fig. S1. Expression of known and novel markers in SOX9iresGFP unchallenged dataset.

A) *Sox2* and *Sox9* were used to identify SCs while genes encoding for hormones (*Gh*, *Prl*, *Tshβ*, *Lhβ*, *Fshβ*, *Pomc*) and lineage specific transcription factors were examined to distinguish committed cells (*Pou1f1* for somatotrophs, lactotrophs and thyrotrophs, *Tbx19* and *Pax7* for corticotrophs and melanotrophs, and *Nr5a1* for gonadotrophs). B) Heatmap for the top10cluster markers.

Sup. Fig.2

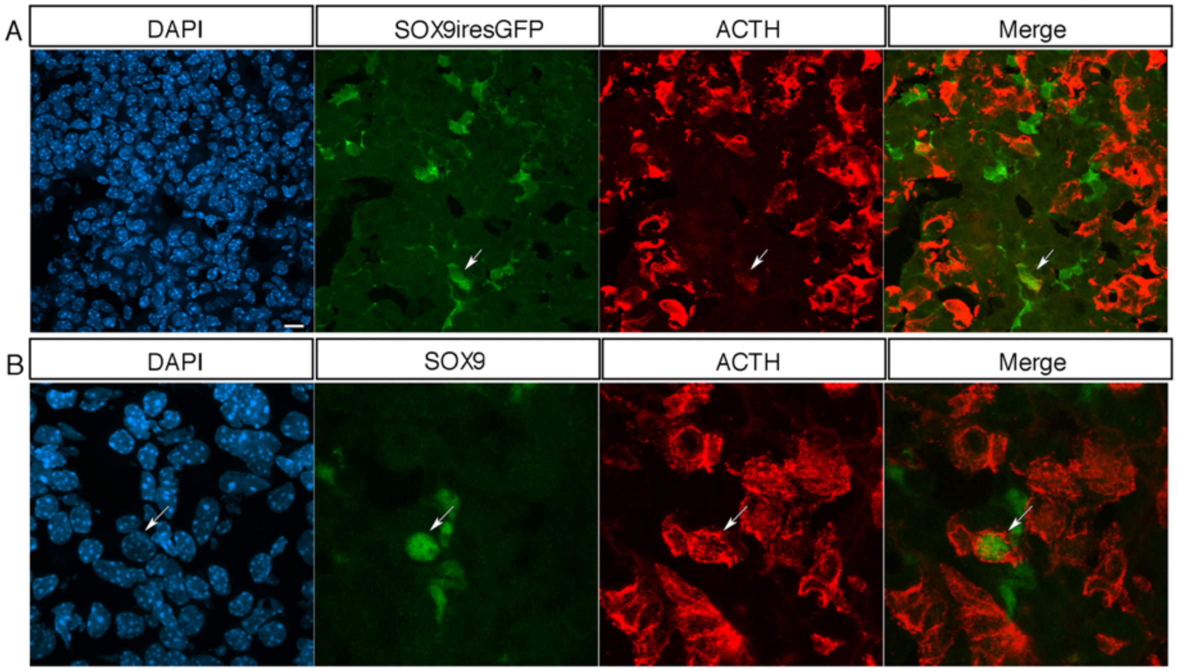

Fig. S2. Co-localisation between SOX9 and ACTH after Ax.

Co-immunofluorescence for GFP, SOX9 and ACTH on sections of pituitaries harvested one week after Ax. Expression of Sox9iresGFP (A) and SOX9 (B) is observed in rare ACTH +ve cells (arrows) exclusively after Ax. The scale bar represents 10 µm for A and 5µm for B.

Sup. Fig.3

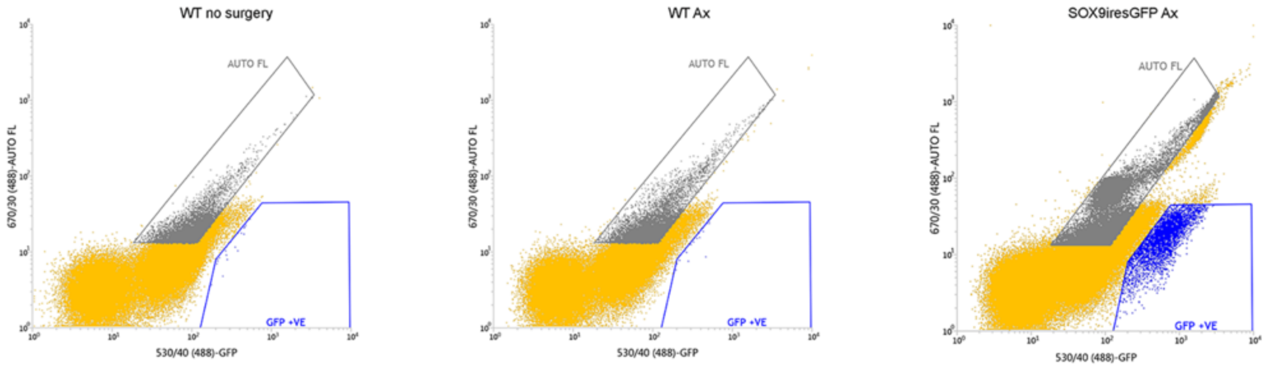

Statistics: 20230126 KR INFLUX A_CONTROL

Populations	Events	% Total	% Parent	FSC Mean	SSC Mean
All Events	124,738	100.00%	####	14,605	11,663
P1 SCATTER GATE	102,436	82.12%	82.12%	16,890	13,578
P2 SINGLET GATE	100,000	80.17%	97.62%	16,840	13,448
GFP +VE	24	0.02%	0.02%	7,634	9,554
AUTO FL	12,098	9.70%	9.70%	20,590	19,283

Statistics: 20230126 KR INFLUX A_SAMPLE Ax

Populations	Events	% Total	% Parent	FSC Mean	SSC Mean
All Events	132,584	100.00%	####	13,028	9,199
P1 SCATTER GATE	102,041	76.96%	76.96%	15,850	11,445
P2 SINGLET GATE	100,000	75.42%	98.00%	15,846	11,393
GFP +VE	23	0.02%	0.02%	22,527	22,377
AUTO FL	11,526	8.69%	8.69%	18,968	15,292

Statistics: 20230126 KR INFLUX A_GFP SAMPLE

Populations	Events	% Total	% Parent	FSC Mean	SSC Mean
All Events	263,026	100.00%	####	13,998	10,498
P1 SCATTER GATE	195,761	74.43%	74.43%	17,485	13,472
P2 SINGLET GATE	192,598	73.22%	98.38%	17,436	13,447
GFP +VE	5,000	1.90%	2.60%	14,537	3,804
AUTO FL	38,839	14.77%	14.77%	19,133	17,443

Fig. S3. Gating strategy for SOX9iresGFP cells.

Gating for SOX9iresGFP cells was validated using wild-type animals. The SOX9iresGFP fraction we selected typically represented 1 to 2% of all cells while 0.02% of all cells were falsely sorted in negative controls. This contamination of likely hormonal cells represents a small proportion of the hormonal cells we detect in our unchallenged Sox9iresGFP fraction (3% of 1.9% representing 0.08% of the whole population).

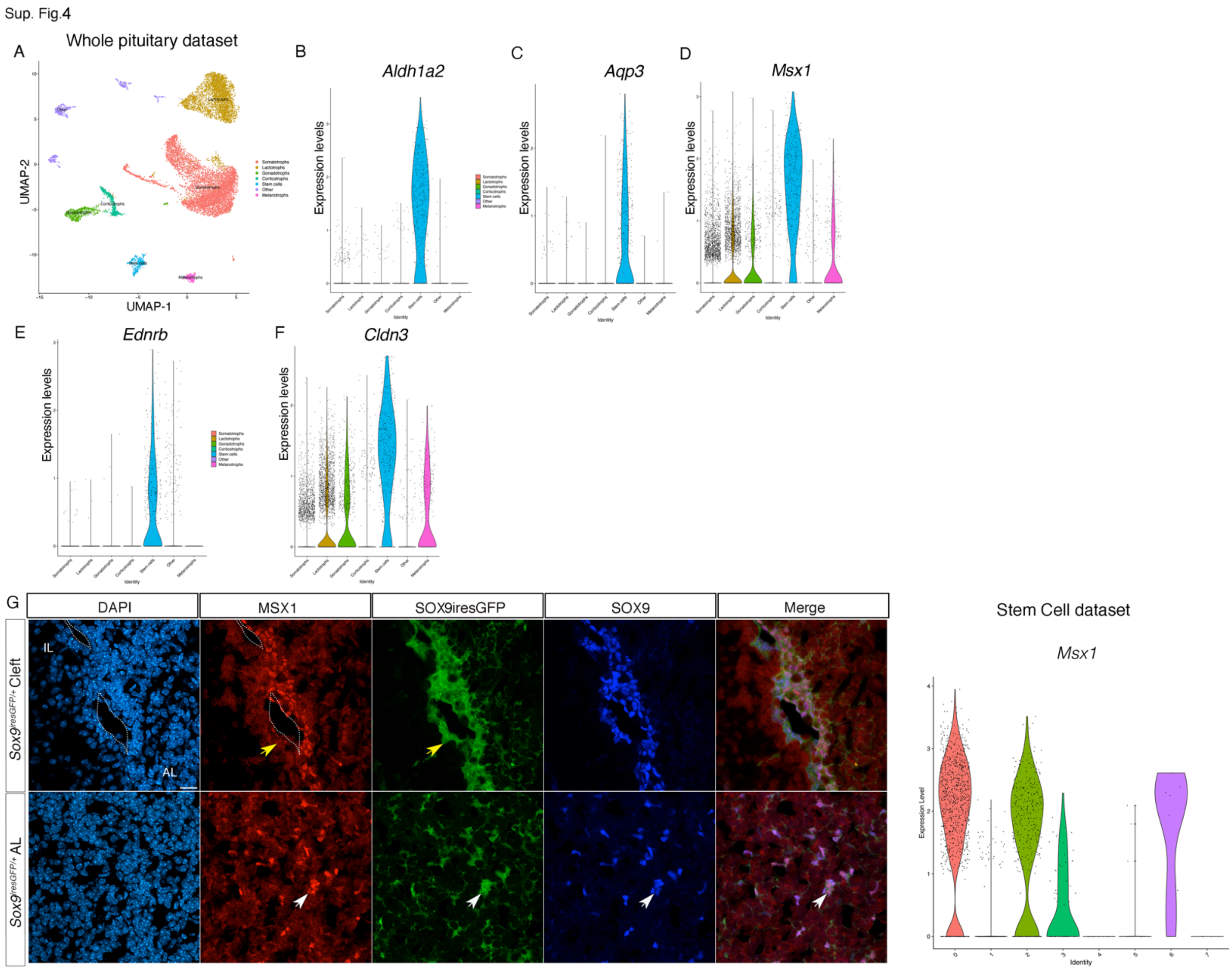

Fig. S4. <u>Expression of novel SC cell markers in whole pituitary and SC datasets.</u>

<u>A) UMAP of re-analysed whole pituitary dataset</u> (*14*). This dataset was used to examine levels of expression of *Ednrb* (B), *Aldah1a2* (C), *Cldn3* (D) and *Msx*1(E) to show exclusive or enriched expression in SCs. F) Co-immunofluorescence for MSX1, GFP and SOX9 in a male SOX9iresGFP pituitary section. MSX1 is exclusively expressed in SOX2;SOX9 AL SC (yellow arrow shows its absence from IL SC, and white arrow shows its expression in parenchymal SC). *Msx1* violin plot shows enrichment in cluster 0 of the single cell dataset presented in Fig.1. The scale bar in A represents 10 µm.

Sup.Fig.5

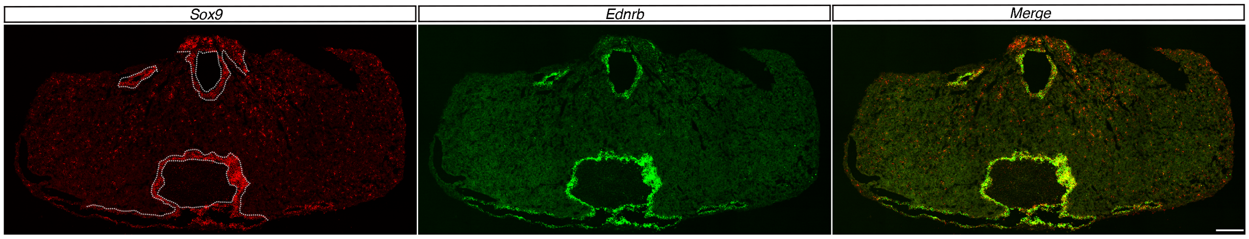

Fig. S5. In situ hybridisation for *Ednrb*.

In situ hybridisation analysis (RNAscope) of *Ednrb* expression confirming higher levels of expression in cleft versus parenchymal SC where low levels of signal can be detected, while levels of *Sox9* expression appear similar in both compartments. The scale bar represents 200 µm. The cleft region is underlined.

Sup. Fig.6

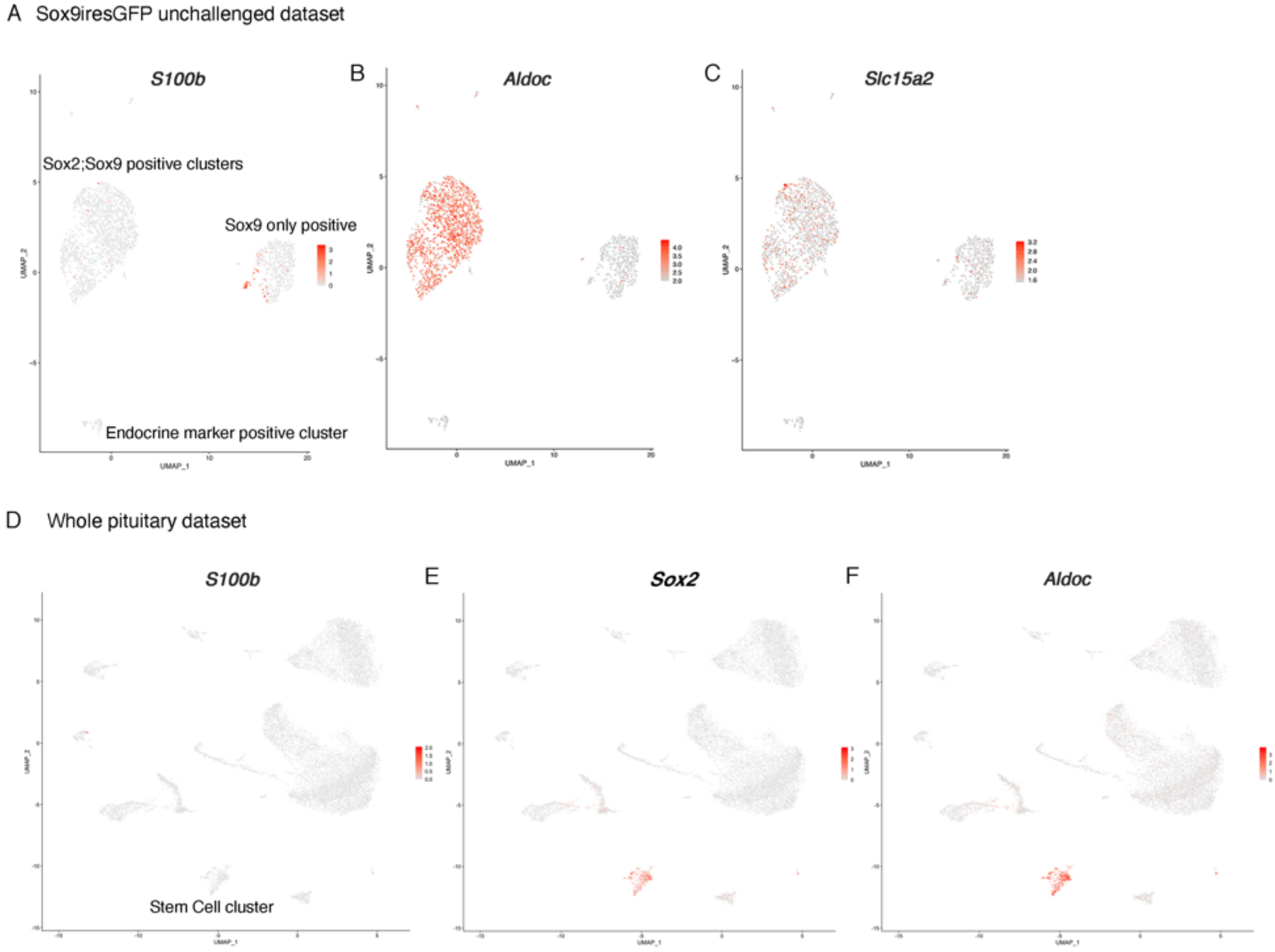

Fig. S6. <u>Expression of FS cells markers in SC and whole pituitary datasets.</u>

A-C) UMAP for *S100b* (A), *Aldolase C* (B) and *Slc15a2* (C) in our Sox9iresGFP single cell dataset.
D-F) UMAP for *S100b* (D), *Sox2* (E) and *Aldolase C* (F) in a whole pituitary dataset (*14*).

Sup. Fig.7

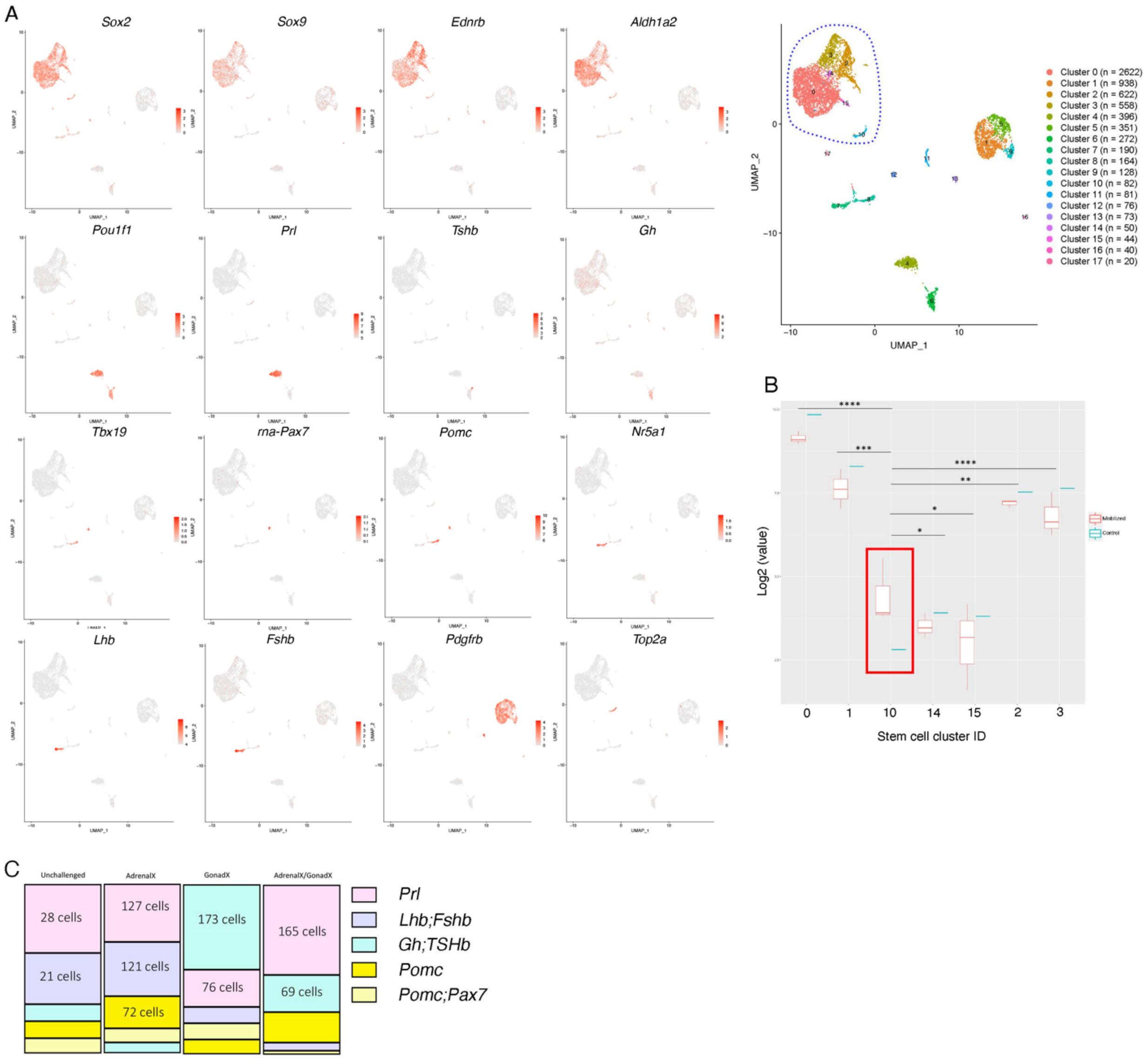

Fig. S7. Unchallenged/Challenged integrated analysis.

A) UMAP clustering and marker analysis for integrated datasets from sorted *Sox9*$^{iresGFP/+}$ cells from unchallenged, adrenalectomized, gonadectomized and both adrenalectomized and gonadectomized animals, 4 days after surgery.

B) Pairwise comparison for proportion test ('pairwise_prop_test' function [stats R package]) on stem cell clusters (circled on UMAP in A). The number of cells assigned to cluster 10 corresponding to proliferative cells (see *Top2a* expression in A) is significantly higher in activated stem cells (Sup. Table2).

Sup. Fig.8

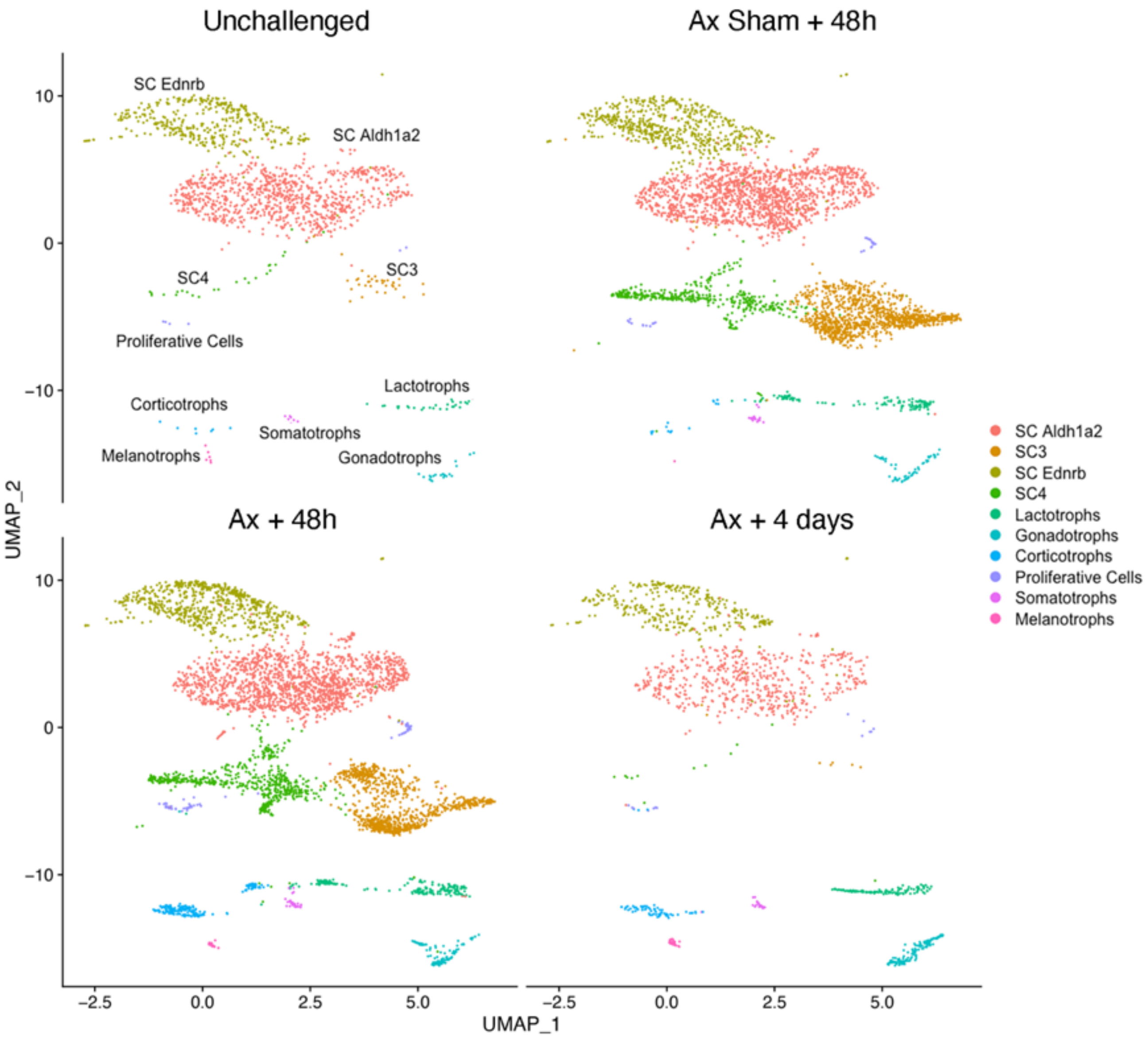

Fig. S8. UMAP clustering for filtered integrated analysis split by dataset.

A) UMAP clustering for integrated datasets from sorted SOX9iresGFP +ve cells from unchallenged, adrenalectomized and sham + 48 h, adrenalectomized + 4 days split by dataset. SC clusters 3 and 4 mostly originate from datasets collected 48 hours after surgery.

Sup. Fig.9

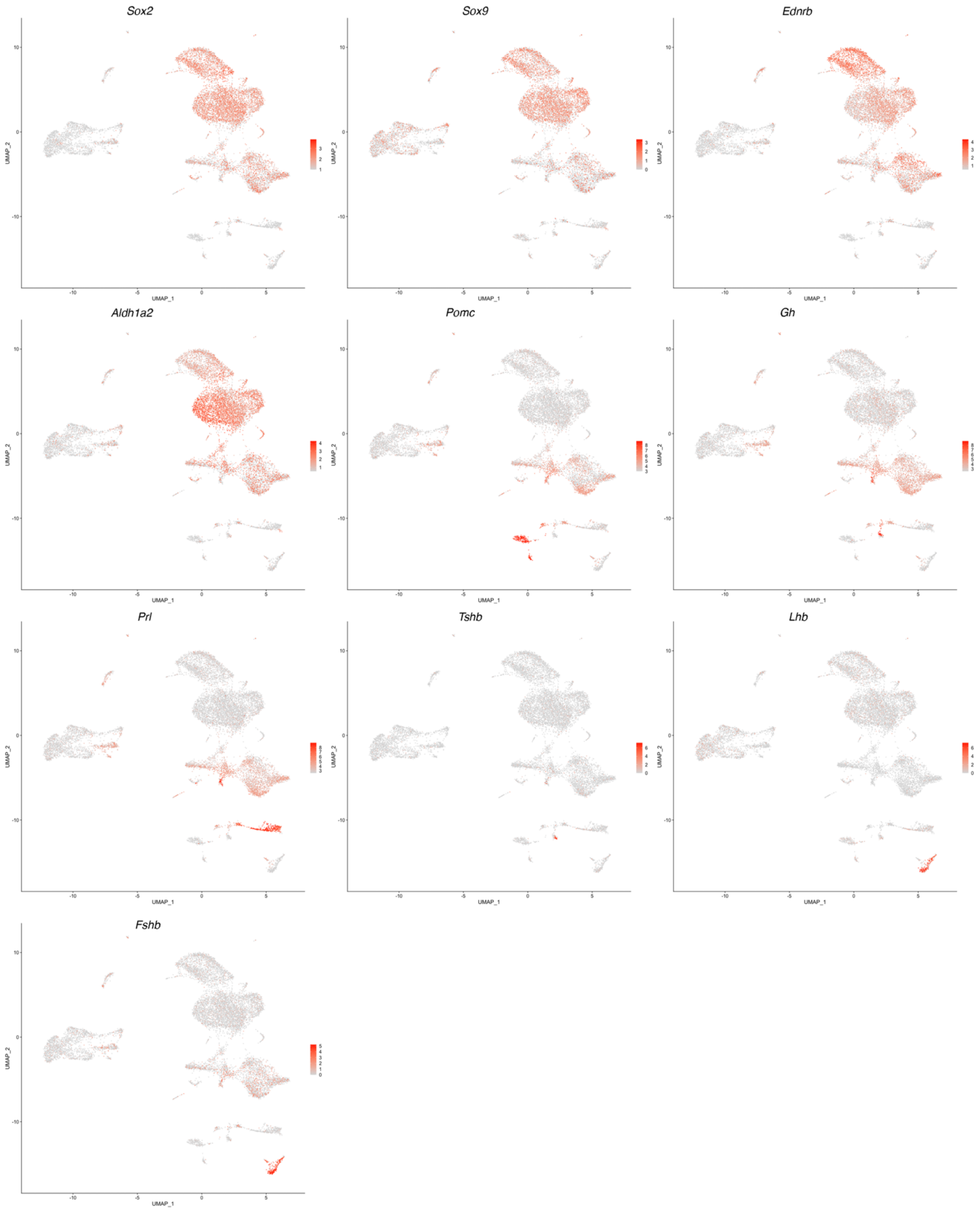

Fig. S9. Analyses of marker expression on UMAP clustering in Ax integrated datasets.

Markers of SC and hormone-secreting cell types are shown on the UMAP clustering for integrated datasets from GFP +ve cells sorted from *Sox9*$^{iresGFP/+}$ pituitaries from mice that were unchallenged, adrenalectomized and sham + 48 h, adrenalectomized + 4 days.

Sup. Fig.10

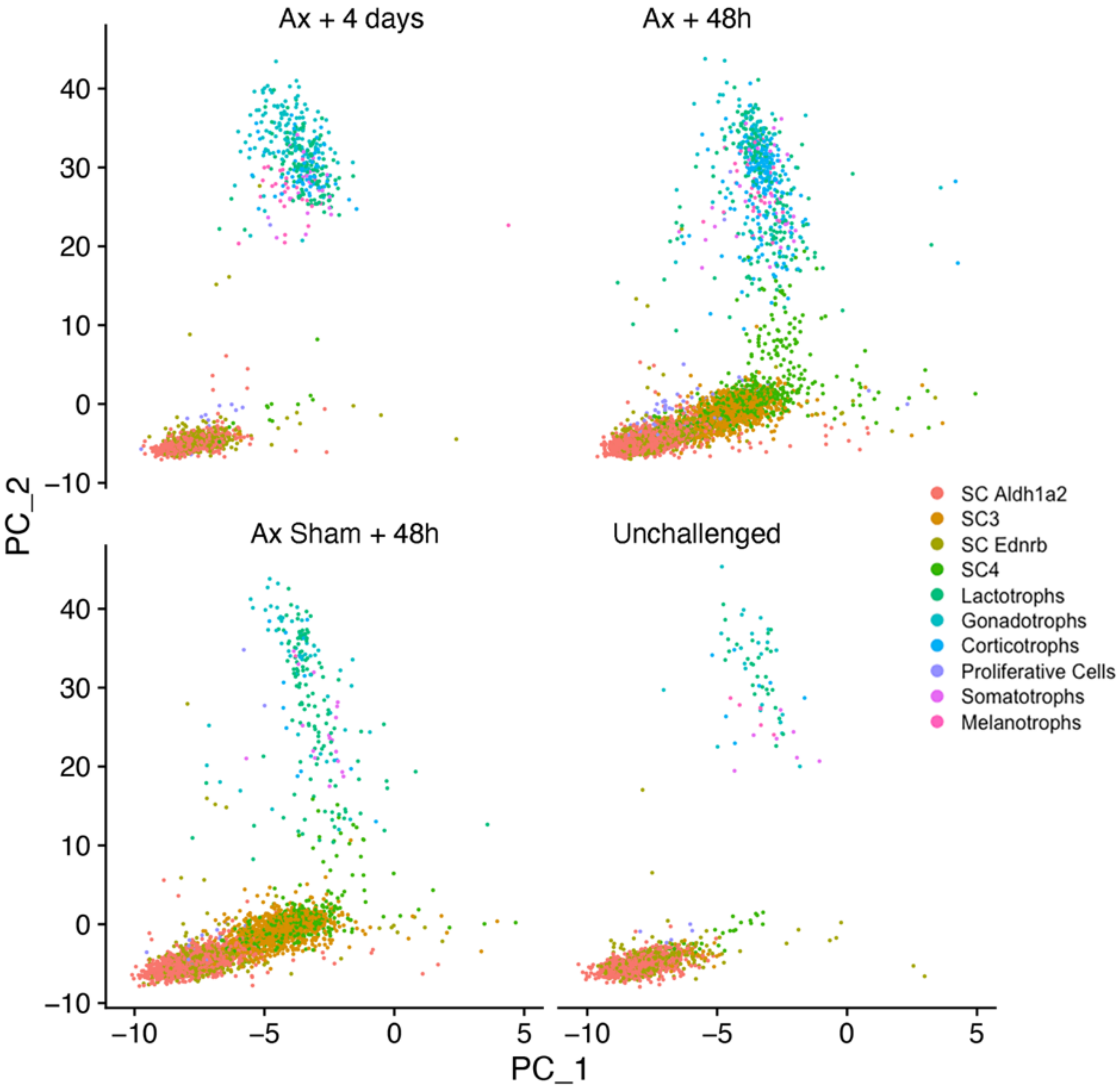

Fig. S10. PCA clustering for filtered integrated analysis split by dataset.

SC4 cluster cells located in an intermediate position between SC and endocrine cells originate mostly from Ax+48h, which is consistent with mobilization and increased commitment toward differentiation.

Sup. Fig.11

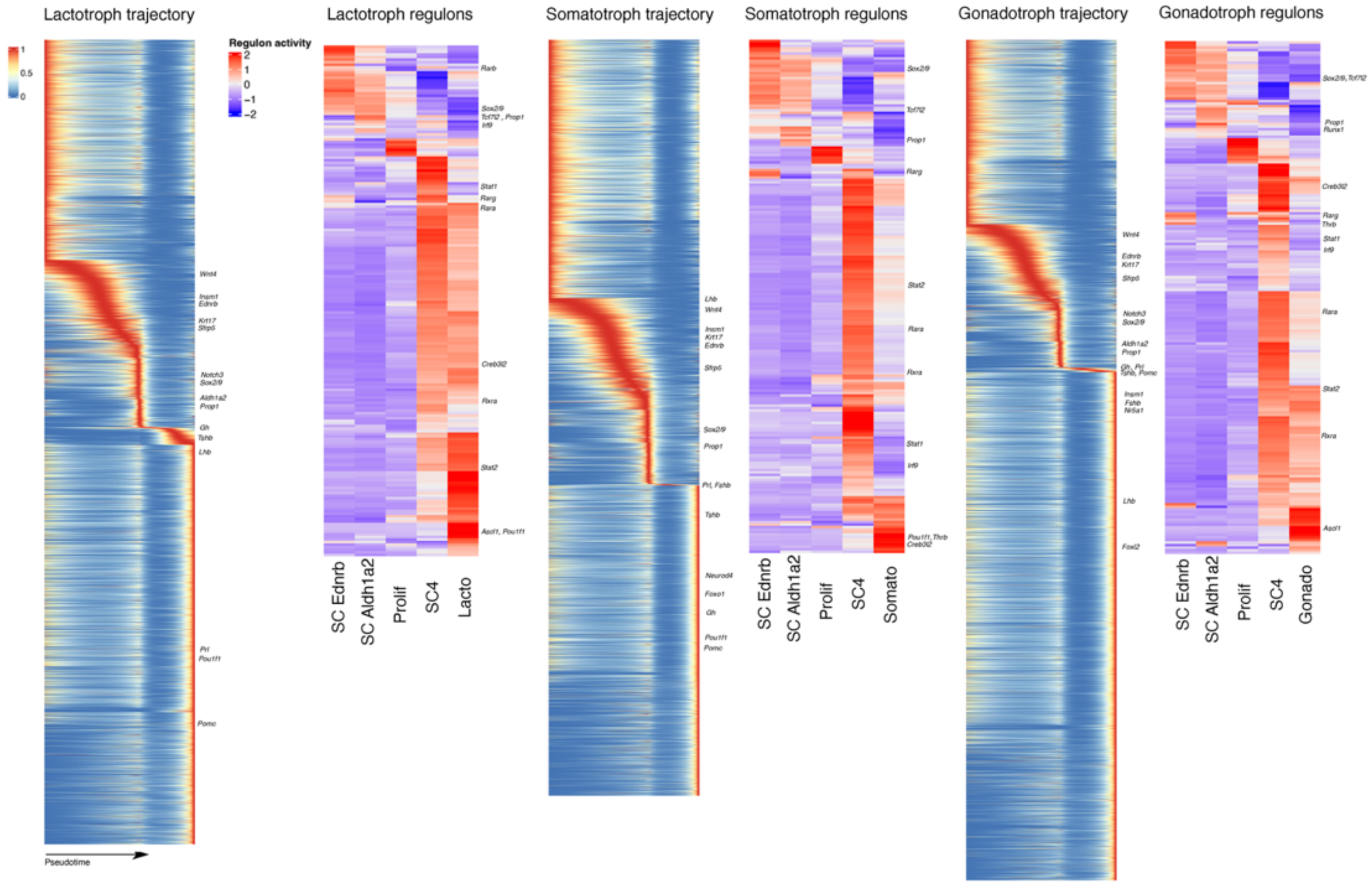

Fig. S11. Analysis of endocrine trajectories and regulons.

Heatmaps of genes associated with trajectories and SCENIC analyses for lactotroph, somatotroph and gonadotroph lineages. Representative genes are shown. Known regulators and those in common with the corticotroph analyses (Fig.5 F-H) are displayed on the heatmaps.

Sup. Fig.12

Whole pituitary dataset

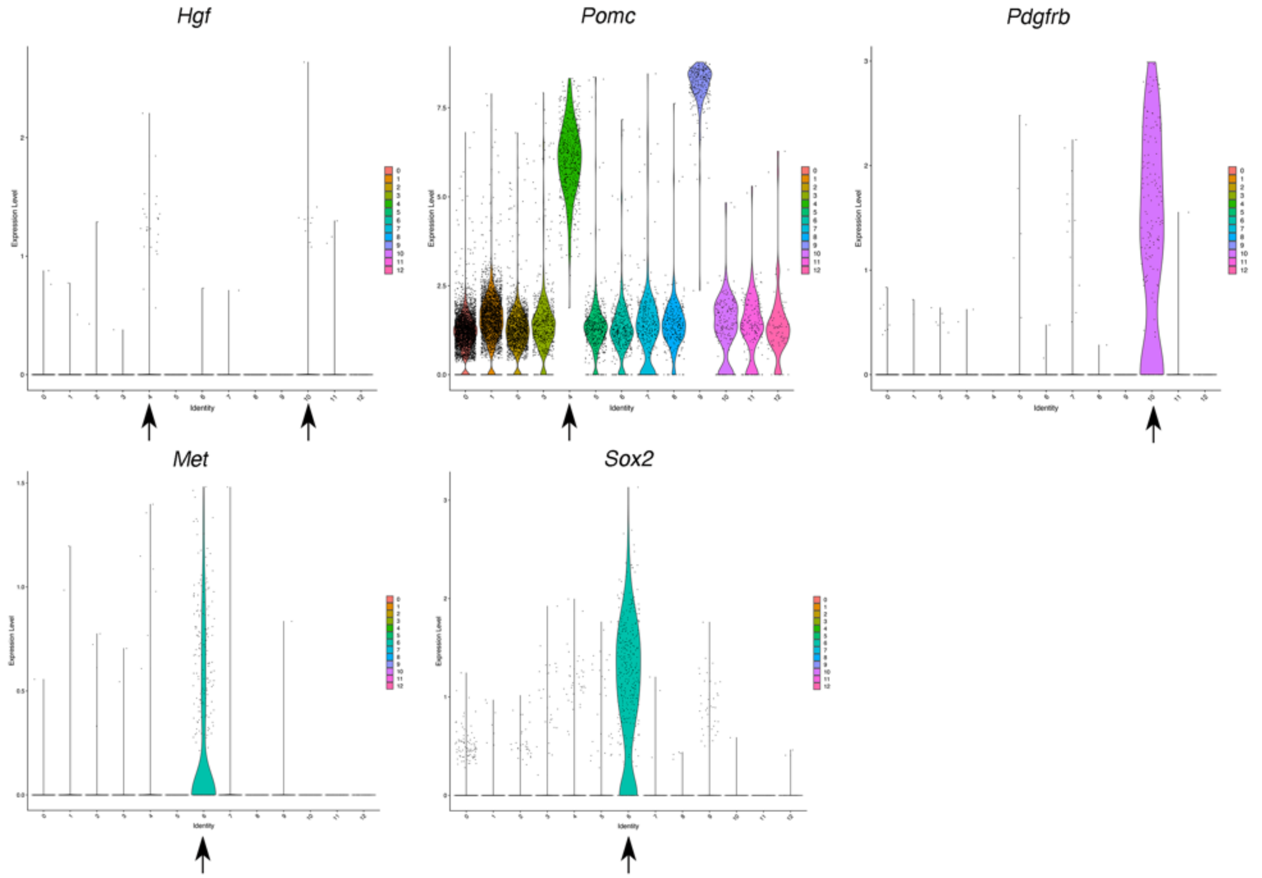

Fig. S12. <u>Expression of *Met* and *Hgf* in the whole pituitary dataset.</u>

Expression of *Hgf* was plotted along with *Pomc* and *Pdgfrb* suggesting expression in *Pomc* positive corticotrophs and *Pdgfrb* positive pericytes, while *Met* expression is restricted to *Sox2* positive SCs (from re-analysed dataset (*28*)). The same pattern is observed in a different dataset (*37*).

Table S1.

Enrichment analysis report

Enrichment by Pathway Maps

#	Maps	Total	pValue	Min FDR	WT Cluster 0 [illegible] p-value	FDR	In Data	Network Objects from Active Data
1	Gamma-secretase proteolytic targets	80	2.041E-11	2.445E-08	2.041E-11	2.445E-08	18	NOTCH2 (2ICD), LRP1B, alphaAPPs, LRP1B (ICD), N-cadherin, NOTCH2 receptor, APP-C99, N-cadherin (CTF1), APP-C83 (CTF), N-cadherin (CTF2), Ephrin-B2, LRP1B (CTF), Ephrin-B2 (CTF2), APP-C59 (AICD), Amyloid beta 40, NOTCH2(NEXT), Amyloid beta 42, APP
2	Development_Extraembryonic differentiation of embryonic stem cells	35	6.983E-07	4.183E-04	6.983E-07	4.183E-04	9	ID2, ID4, HEY1, SOX2, ID1, MAP2, NCAM1, PAX6, CD9
3	Neuroprotective action of lithium	63	2.603E-06	7.638E-04	2.603E-06	7.638E-04	11	FRAT1, Thioredoxin, Amyloid beta, P2X2, IPP-1, NR2A, WNT, NR2, p38 MAPK, Frizzled, c-Jun
4	Development_NOTCH signaling in the nervous system	89	2.634E-06	7.638E-04	2.634E-06	7.638E-04	13	NOTCH2 (2ICD), ID4, HEY1, SOX2, NOTCH2 receptor, Aquaporin 4, HES1, Adropin, Cyclin D1, NFIA, PAX6, NOTCH2(NEXT), Clusterin
5	Development_NOTCH signaling in organogenesis and embryogenesis	77	3.188E-06	7.638E-04	3.188E-06	7.638E-04	12	SIX2, NOTCH2 (2ICD), IRF6, HEY1, NOTCH2 receptor, HES1, Islet-1, HEY2, Ephrin-B2, Activin beta A, Connexin 43, NRARP
6	Inhibition of oligodendrocyte precursor cells differentiation by Wnt signaling in multiple sclerosis	25	6.731E-06	1.220E-03	6.731E-06	1.220E-03	7	FZD1, ID2, WNT9A, FZD7, WNT, Frizzled, FZD4
7	Gamma-Secretase regulation of neuronal cell development and function	57	7.131E-06	1.220E-03	7.131E-06	1.220E-03	10	N-cadherin, Amyloid beta, APP-C99, Fe65, N-cadherin (CTF1), N-cadherin (CTF2), APP-C59 (AICD), Amyloid beta 40, betaAPPs, APP
8	Gamma-secretase regulation of gastrointestinal epithelial cell development	28	1.532E-05	2.295E-03	1.532E-05	2.295E-03	7	NOTCH2 (2ICD), HEY1, NOTCH2 receptor, HES1, HEY2, PAX6, NOTCH2(NEXT)
9	Role of IGH translocations in multiple myeloma	40	2.198E-05	2.925E-03	2.198E-05	2.925E-03	8	MafB, N-cadherin, NOTCH2 receptor, ID1, HES1, Cyclin D1, NCAM1, Histone H3
10	Inhibition of Ephrin receptors in colorectal cancer	30	2.500E-05	2.995E-03	2.500E-05	2.995E-03	7	Ephrin-B2, Ephrin-B, WNT, Ephrin-B receptors, Ephrin-A receptors, Frizzled, Ephrin-B receptor 1
11	Deregulation of canonical WNT signaling in major depressive disorder	31	3.146E-05	3.193E-03	3.146E-05	3.193E-03	7	FRAT1, FZD3, WNT, Cyclin D1, FZD9, MAP-1B, Frizzled
12	Transcription targets of Androgen receptor involved in Prostate Cancer	42	3.198E-05	3.193E-03	3.198E-05	3.193E-03	8	Androgen receptor, N-cadherin, ER81, Cyclin D1, Prosaposin, VIL2 (ezrin), Clusterin, APP
13	Cell adhesion_Gap junctions	22	3.713E-05	3.357E-03	3.713E-05	3.357E-03	6	Tubulin beta, Actin cytoskeletal, Actin, Calmodulin, Connexin 43, VIL2 (ezrin)
14	Stem cells_Notch signaling in medulloblastoma stem cells	32	3.923E-05	3.357E-03	3.923E-05	3.357E-03	7	NOTCH2 (2ICD), HEY1, NOTCH2 receptor, HES1, HEY2, Cyclin D1, NOTCH2(NEXT)
15	Role of activation of WNT signaling in the progression of lung cancer	77	1.056E-04	8.437E-03	1.056E-04	8.437E-03	10	FZD1, FZD3, WNT9A, FZD7, WNT, Cyclin D1, p38 MAPK, FZD9, CD147, Frizzled
16	Signal transduction_NOTCH signaling in tissue homeostasis	81	1.624E-04	1.195E-02	1.624E-04	1.195E-02	10	NOTCH2 (2ICD), Androgen receptor, HEY1, NOTCH2 receptor, HES1, HEY2, Ephrin-B2, c-Jun, OAT, CYP19
17	WNT signaling in HCC	40	1.763E-04	1.195E-02	1.763E-04	1.195E-02	7	FZD3, Ep-CAM, FZD7, WNT, Cyclin D1, GLNA, Frizzled
18	Immune response_Induction of the antigen presentation machinery by IFN-gamma	53	1.795E-04	1.195E-02	1.795E-04	1.195E-02	8	H2-Aa, H2-Eb1, HLA-E, MHC class II, HLA-F, HLAC, CD74, HLA-DMA
19	Immune response_Antigen presentation by MHC class II	118	2.422E-04	1.527E-02	2.422E-04	1.527E-02	12	MHC class II alpha chain, HSP90, Kinesin light chain, MHC class II beta chain, Dynein 1, cytoplasmic, intermediate chains, HLA-DM, MHC class II, p38 MAPK, HSP90 beta, CLEC10A, Legumain, CD74
20	Glutathione metabolism	71	2.809E-04	1.683E-02	2.809E-04	1.683E-02	9	GCL cat, GSTM1, GSTA3, GSTM2, GSTM5, MGST, GGTL3, GSTM1 (rodent), GSTT1
21	Oxidative stress_Role of Sirtuin1 and PGC1-alpha in activation of antioxidant defense system	60	4.308E-04	2.458E-02	4.308E-04	2.458E-02	8	GCL cat, SCPX, Thioredoxin, PRDX1, TXNRD1, GSTM5, UCP2, PRDX5
22	Development_TGF-beta-dependent induction of EMT via SMADs	35	5.787E-04	3.126E-02	5.787E-04	3.126E-02	6	ID2, HEY1, N-cadherin, TGF-beta 2, TGF-beta, MKL1
23	Transcription_HIF-1 targets	95	6.001E-04	3.126E-02	6.001E-04	3.126E-02	10	ID2, SOX2, TGF-beta 2, P4HA1, ALDOC, DEC2, ENO1, REDD1, PKM2, GPI
24	Beta-catenin-dependent transcription regulation in colorectal cancer	36	6.769E-04	3.379E-02	6.769E-04	3.379E-02	6	ID2, Cyclin D1, NRCAM, LAMC2, LAMC2 (80kDa), LAMC2 (100kDa)
25	Development_NOTCH signaling activation	82	8.250E-04	3.953E-02	8.250E-04	3.953E-02	9	NOTCH2 (2ICD), HEY1, NOTCH2 receptor, HES1, HEY2, BAF60c, HDAC3, MSI1, NOTCH2(NEXT)
26	Canonical Notch signaling pathway in colorectal cancer	52	9.256E-04	4.123E-02	9.256E-04	4.123E-02	7	NOTCH2 (2ICD), NOTCH2 receptor, HES1, WNT, Cyclin D1, NOTCH2(NEXT), HES6
27	Cell adhesion_Endothelial cell contacts by junctional mechanisms	26	9.751E-04	4.123E-02	9.751E-04	4.123E-02	5	N-cadherin, Actin cytoskeletal, Cingulin, Alpha-catenin, Connexin 43
28	Cell adhesion_Classical cadherin-mediated cell adhesion	26	9.751E-04	4.123E-02	9.751E-04	4.123E-02	5	N-cadherin, Actin cytoskeletal, F-Actin cytoskeleton, Alpha-catenin, F-Actin
29	Cell cycle progression in Prostate Cancer	39	1.050E-03	4.123E-02	1.050E-03	4.123E-02	6	Androgen receptor, WNT, Cyclin D1, p38 MAPK, Frizzled, c-Jun
30	Activation of Notch signaling in breast cancer	39	1.050E-03	4.123E-02	1.050E-03	4.123E-02	6	HEY1, HES1, p38 MAPK, c-Jun, MSI1, Presenilin
31	Transcription_Negative regulation of HIF1A function	69	1.111E-03	4.123E-02	1.111E-03	4.123E-02	8	HSP90, PRDX2, SKP1, DEC2, HSP90 beta, KLF2, HIF-prolyl hydroxylase, EGLN2
32	NRF2 regulation of oxidative stress response	54	1.163E-03	4.123E-02	1.163E-03	4.123E-02	7	GCL cat, GSTA3, Thioredoxin, PRDX1, TXNRD1, Actin cytoskeletal, CRM1
33	Stem cells_NOTCH1-induced self-renewal of glioblastoma stem cells	40	1.205E-03	4.123E-02	1.205E-03	4.123E-02	6	HEY1, SOX2, ID1, HES1, HEY2, Cyclin D1
34	Gamma-secretase regulation of osteogenesis	40	1.205E-03	4.123E-02	1.205E-03	4.123E-02	6	NOTCH2 (2ICD), HEY1, NOTCH2 receptor, HES1, Cyclin D1, NOTCH2(NEXT)
35	Stem cells_Response to hypoxia in glioblastoma stem cells	40	1.205E-03	4.123E-02	1.205E-03	4.123E-02	6	HEY1, ID1, HES1, HEY2, Cyclin D1, HIF-prolyl hydroxylase
36	DNA damage_ATM activation by DNA damage	71	1.341E-03	4.343E-02	1.341E-03	4.343E-02	8	HSP90, HMG14, p18, PP2A regulatory, HSP90 beta, Histone H2B, IHPK2, Histone H3
37	Development_Positive regulation of STK3/4 (Hippo) pathway and negative regulation of YAP/TAZ func	71	1.341E-03	4.343E-02	1.341E-03	4.343E-02	8	EBP50, Angiomotin (AMOT), Actin cytoskeletal, MALS-3, Alpha-catenin, Alpha-1 catenin, G-protein alpha-s, 14-3-3
38	G-protein signaling_Rac3 regulation pathway	17	1.453E-03	4.580E-02	1.453E-03	4.580E-02	4	Androgen receptor, Actin cytoplasmic 2, Calmyrin, F-Actin
39	Ubiquinone metabolism	74	1.756E-03	5.333E-02	1.756E-03	5.333E-02	8	MTND1, NDUFA4, MT-ND5, MTND4, MTND2, NDUFC2, DAP13, MTND3
40	Notch signaling in breast cancer	58	1.781E-03	5.333E-02	1.781E-03	5.333E-02	7	NOTCH2 (2ICD), HEY1, NOTCH2 receptor, HES1, HEY2, Cyclin D1, NOTCH2(NEXT)
41	Androgen receptor activation and downstream signaling in Prostate cancer	110	1.865E-03	5.450E-02	1.865E-03	5.450E-02	10	Androgen receptor, N-cadherin, ER81, SGK1, Cyclin D1, Prosaposin, FGF1, VIL2 (ezrin), Clusterin, APP
42	Development_Transcription factors in segregation of hepatocytic lineage	30	1.912E-03	5.454E-02	1.912E-03	5.454E-02	5	Activin A, Activin B, Cyclin D1, Activin, GGTL3
43	Aminoglycoside- and cisplatin-induced hair cell death	112	2.134E-03	5.749E-02	2.134E-03	5.749E-02	10	NOL3, AP-1, p38 MAPK, HDAC3, Histone H2B, Cathepsin D, CX3CL1, c-Jun, Histone H3, LMO4
44	Role of inhibition of WNT signaling in the progression of lung cancer	31	2.224E-03	5.749E-02	2.224E-03	5.749E-02	5	N-cadherin, CD82, FZD9, Frizzled, c-Jun
45	Development_Embryonal epaxial myogenesis	31	2.224E-03	5.749E-02	2.224E-03	5.749E-02	5	FZD1, HES1, WNT, Pitx2, Frizzled
46	Development_Negative regulation of WNT/Beta-catenin signaling at the receptor level	45	2.251E-03	5.749E-02	2.251E-03	5.749E-02	6	LRP4, Amyloid beta, WNT, Frizzled, FZD4, Syndecans
47	Inhibition of WNT5A-dependent non-canonical pathway in colorectal cancer	19	2.255E-03	5.749E-02	2.255E-03	5.749E-02	4	Cyclin D1, Calmodulin, CRM1, c-Jun
48	Neurophysiological process_ACM1, ACM3 and ACM5 signaling in the brain	78	2.461E-03	6.141E-02	2.461E-03	6.141E-02	8	alphaAPPs, TRPM3, APP-C99, APP-C83 (CTF), Calmodulin, betaAPPs, CACNB3, APP
49	Inhibition of remyelination in multiple sclerosis: role of cell-cell and ECM-cell interactions	47	2.820E-03	6.894E-02	2.820E-03	6.894E-02	6	CD81, Actin cytoskeletal, CD82, NCAM1, Connexin 43, CD9
50	Role of SHH and Notch in SCLC	33	2.956E-03	6.944E-02	2.956E-03	6.944E-02	5	NOTCH2 (2ICD), NOTCH2 receptor, HES1, AL1A1, NOTCH2(NEXT)

Enrichment analysis report

Enrichment by Pathway Maps

#	Maps	Total	pValue	Min FDR	WT Cluster 2 genelist p-value	FDR	In Data	Network Objects from Active Data
1	Cytoskeleton remodeling_Keratin filaments	36	5.894E-09	4.697E-06	5.894E-09	4.697E-06	8	Keratin 19, Keratin 17, Tubulin alpha, Keratin 8, Keratin 8/18, Vimentin, Keratin 18, Keratin 7
2	Cytoskeleton remodeling_Regulation of actin cytoskeleton organization by the kinase effectors of Rho	58	3.015E-07	1.202E-04	3.015E-07	1.202E-04	8	Rac3, MLCP (cat), Rhov, Spectrin, MRLC, Rac1-related, WRCH-1, Cdc42 subfamily
3	Cell adhesion_Desmosomes	19	1.935E-06	5.139E-04	1.935E-06	5.139E-04	5	Keratin 17, Keratin 8/18, Vimentin, Keratin 18, Plakoglobin
4	Cell adhesion_Tight junctions	44	1.038E-05	1.891E-03	1.038E-05	1.891E-03	6	PKC-zeta, Tubulin alpha, Occludin, MRLC, CRB3, ZO-3
5	Mechanisms of resistance to EGFR inhibitors in lung cancer	45	1.186E-05	1.891E-03	1.186E-05	1.891E-03	6	HSP90, Claudin-7, Ep-CAM, Vimentin, Claudin-4, TACSTD2 (TROP2)
6	Canonical Notch signaling pathway in colorectal cancer	52	2.775E-05	3.687E-03	2.775E-05	3.687E-03	6	KLF4, NOTCH1 (NICD), NOTCH1 (NEXT), KLF5, NOTCH1 receptor, NOTCH1 precursor
7	Oxidative stress_ROS-induced cellular signaling	108	3.412E-05	3.885E-03	3.412E-05	3.885E-03	8	HSP27, NOTCH1 (NICD), TXNIP (VDUP1), FTH1, NFKBIA, Pin1, DLC1 (Dynein LC8a), PKC
8	Possible regulation of HSF-1/ chaperone pathway in Huntington's disease	21	8.687E-05	8.655E-03	8.687E-05	8.655E-03	4	HSP90, HSP27, PLA2, HSP90 alpha
9	Signal transduction_Relaxin family peptides signaling via RXFP1 and RXFP2 receptors	70	1.508E-04	1.335E-02	1.508E-04	1.335E-02	6	NOTCH1 (NICD), PKC-zeta, NOTCH1 (NEXT), EDNRB, NFKBIA, I-kB
10	Cytoskeleton remodeling_Regulation of actin cytoskeleton nucleation and polymerization by Rho GTP	46	1.776E-04	1.415E-02	1.776E-04	1.415E-02	5	Rac3, mDIA2(DIAPH3), Rac1-related, DRF, Cdc42 subfamily
11	Cell adhesion_Endothelial cell contacts by junctional mechanisms	26	2.075E-04	1.503E-02	2.075E-04	1.503E-02	4	Occludin, Vimentin, Claudin-3, Plakoglobin
12	Glucocorticoids-mediated inhibition of pro-constrictory and pro-inflammatory signaling in airway smo	49	2.403E-04	1.596E-02	2.403E-04	1.596E-02	5	MLCP (cat), PLA2, NFKBIA, MRLC, MKP-1
13	Notch signaling in oligodendrocyte precursor cell differentiation in multiple sclerosis	29	3.209E-04	1.968E-02	3.209E-04	1.968E-02	4	NOTCH1 (NICD), NOTCH1 (NEXT), SOX10, NOTCH1 receptor
14	Development_Auditory hair cell differentiation in embryogenesis	31	4.176E-04	2.378E-02	4.176E-04	2.378E-02	4	NOTCH1 (NICD), NOTCH1 (NEXT), NOTCH1 receptor, NOTCH1 precursor
15	Skeletal muscle atrophy in COPD	57	4.901E-04	2.604E-02	4.901E-04	2.604E-02	5	NFKBIA, I-kB, Vimentin, 14-3-3, JunD
16	Notch signaling in breast cancer	58	5.314E-04	2.620E-02	5.314E-04	2.620E-02	5	NOTCH1 (NICD), NOTCH1 (NEXT), Pin1, NOTCH1 receptor, NOTCH1 precursor
17	Development_NOTCH signaling in the nervous system	89	5.589E-04	2.620E-02	5.589E-04	2.620E-02	6	NOTCH1 (NICD), PKC-zeta, NOTCH1 (NEXT), SOX10, NOTCH1 receptor, Clusterin
18	Development_WNT and Notch signaling in early cardiac myogenesis	35	6.706E-04	2.969E-02	6.706E-04	2.969E-02	4	NOTCH1 (NICD), SFRP5, NOTCH1 (NEXT), NOTCH1 receptor
19	Immune response_IL-4 signaling pathway	94	7.471E-04	2.995E-02	7.471E-04	2.995E-02	6	AP-1, PKC-zeta, Tubulin alpha, NFKBIA, PKC, JunD
20	Cytoskeleton remodeling_Substance P mediated membrane blebbing	16	7.515E-04	2.995E-02	7.515E-04	2.995E-02	3	MLCP (cat), Tubulin alpha, MRLC
21	Stem cells_CD30 signaling in transformed embryonic stem cells	18	1.077E-03	4.087E-02	1.077E-03	4.087E-02	3	KLF4, I-kB, TRAF1
22	Development_NOTCH-induced EMT	19	1.268E-03	4.593E-02	1.268E-03	4.593E-02	3	NOTCH1 (NICD), NOTCH1 (NEXT), NOTCH1 receptor
23	Signal transduction_Non-neuronal ACM1, ACM3 and ACM5 signaling	74	1.613E-03	5.588E-02	1.613E-03	5.588E-02	5	MLCP (cat), PKC-zeta, MRLC2, MRLC, PKC
24	Role of DNA methylation in progression of multiple myeloma	45	1.746E-03	5.797E-02	1.746E-03	5.797E-02	4	AP-1, SFRP5, DKK3, NOTCH1 receptor
25	Signal transduction_Angiotensin II/AGTR1 signaling via Notch, Beta-catenin and NF-kB pathways	78	2.037E-03	6.495E-02	2.037E-03	6.495E-02	5	NOTCH1 (NICD), NOTCH1 (NEXT), I-kB, NOTCH1 receptor, PKC
26	Development_NOTCH signaling activation	82	2.538E-03	7.781E-02	2.538E-03	7.781E-02	5	NOTCH1 (NICD), PKC-zeta, NOTCH1 (NEXT), NOTCH1 receptor, NOTCH1 precursor
27	HSP70 and HSP40-dependent folding in Huntington's disease	25	2.861E-03	8.446E-02	2.861E-03	8.446E-02	3	HSP90, HSP27, HSP90 alpha
28	Signal transduction_Additional pathways of NF-kB activation (in the cytoplasm)	52	2.981E-03	8.485E-02	2.981E-03	8.485E-02	4	PKC-zeta, NFKBIA, Pin1, I-kB
29	Gamma-secretase regulation of gastrointestinal epithelial cell development	28	3.974E-03	1.056E-01	3.974E-03	1.056E-01	3	NOTCH1 (NICD), NOTCH1 (NEXT), NOTCH1 receptor
30	Rho-dependent regulation of normal and asthmatic smooth muscle contraction	28	3.974E-03	1.056E-01	3.974E-03	1.056E-01	3	MLCP (cat), MRLC, PKC
31	Development_VEGF signaling via VEGFR2 - generic cascades	93	4.372E-03	1.070E-01	4.372E-03	1.070E-01	5	HSP90, HSP27, I-kB, PKC, PLA2G5
32	Cigarette smoke-mediated regulation of NRF2-antioxidant pathway in airway epithelial cells	29	4.396E-03	1.070E-01	4.396E-03	1.070E-01	3	GSTO1, TALDO, DJ-1
33	Aberrant production of IL-2 and IL-17 in SLE T cells	58	4.430E-03	1.070E-01	4.430E-03	1.070E-01	4	NOTCH1 (NICD), AP-1, I-kB, NOTCH1 precursor
34	Gamma-secretase regulation of angiogenesis	30	4.843E-03	1.103E-01	4.843E-03	1.103E-01	3	NOTCH1 (NICD), NOTCH1 (NEXT), NOTCH1 receptor
35	Signal transduction_Additional pathways of NF-kB activation (in the nucleus)	30	4.843E-03	1.103E-01	4.843E-03	1.103E-01	3	PKC-zeta, NFKBIA, I-kB
36	Role of inhibition of WNT signaling in the progression of lung cancer	31	5.317E-03	1.177E-01	5.317E-03	1.177E-01	3	Keratin 8, Vimentin, Keratin 18
37	Stem cells_Notch signaling in medulloblastoma stem cells	32	5.818E-03	1.253E-01	5.818E-03	1.253E-01	3	NOTCH1 (NICD), NOTCH1 (NEXT), NOTCH1 receptor
38	Role of SHH and Notch in SCLC	33	6.347E-03	1.292E-01	6.347E-03	1.292E-01	3	NOTCH1 (NICD), NOTCH1 (NEXT), NOTCH1 receptor
39	LRRK2 in neurons in Parkinson's disease	33	6.347E-03	1.292E-01	6.347E-03	1.292E-01	3	HSP90, PKC-zeta, 14-3-3
40	Development_NOTCH1 signaling in the epidermis	65	6.645E-03	1.292E-01	6.645E-03	1.292E-01	4	NOTCH1 (NICD), NOTCH1 (NEXT), I-kB, NOTCH1 receptor
41	Signal transduction_PDGF signaling via MAPK cascades	65	6.645E-03	1.292E-01	6.645E-03	1.292E-01	4	HSP27, KLF4, AP-1, JunD
42	Development_Epigenetic and transcriptional regulation of oligodendrocyte precursor cell differentiatior	34	6.903E-03	1.310E-01	6.903E-03	1.310E-01	3	NOTCH1 (NICD), SOX10, NOTCH1 receptor
43	Development_TGF-beta-dependent induction of EMT via SMADs	35	7.488E-03	1.356E-01	7.488E-03	1.356E-01	3	NOTCH1 (NICD), Occludin, Vimentin
44	Immune response_Lipoxins and Resolvin E1 inhibitory action on neutrophil functions	35	7.488E-03	1.356E-01	7.488E-03	1.356E-01	3	PKC-zeta, NFKBIA, I-kB
45	HBV signaling via protein kinases leading to HCC	36	8.100E-03	1.435E-01	8.100E-03	1.435E-01	3	AP-1, Pin1, PKC
46	Immune response_Plasmin signaling	70	8.612E-03	1.492E-01	8.612E-03	1.492E-01	4	Annexin II, NFKBIA, PKC, JunD
47	TNF-alpha-induced inflammatory signaling in normal and asthmatic airway epithelium	38	9.412E-03	1.576E-01	9.412E-03	1.576E-01	3	NFKBIA, FN14(TNFRSF12A), I-kB
48	Signal transduction_Calcium-mediated signaling	72	9.494E-03	1.576E-01	9.494E-03	1.576E-01	4	MLCP (cat), I-kB, PKC, 14-3-3
49	Activation of Notch signaling in breast cancer	39	1.011E-02	1.630E-01	1.011E-02	1.630E-01	3	KLF4, NOTCH1 (NICD), NOTCH1 precursor
50	Gamma-secretase regulation of osteogenesis	40	1.084E-02	1.630E-01	1.084E-02	1.630E-01	3	NOTCH1 (NICD), NOTCH1 (NEXT), NOTCH1 receptor

Pathway analysis for clusters 0 and 2.

Gene markers for WT Cluster 0 were used to determine enriched pathways with Metacore using a hypergeometric test. Pathways with an adjusted p value < 0.05 are shown in the table. Genes present in our dataset are highlighted in the top5 pathways.

Table S2.

		Number of cells				
ID	Cluster ID	AX+ 4days	AX+ 48h00	AXSham	WT	
SC Aldh1a2	0	541	1606	1492	910	
SC3	1	7	941	1068	36	
SC Ednrb	2	334	685	593	409	
Dcn	3	427	403	493	484	
SC4	4	12	603	387	27	
Dcn/vasc	5	7	212	237	24	
Lacto	6	127	198	120	28	
Gonado	7	136	94	56	22	
Cortico	8	69	199	14	7	
Dcn/vasc	9	22	72	86	21	
Prolif	10	13	69	26	6	
Somato	11	24	37	18	7	
Melano	12	31	33	1	6	
Dcn/vasc	13	0	25	13	5	
Dcn/vasc	14	15	18	4	1	
		1765	5195	4608	1993	
Endo cells total		387	561	209	70	
%endo/total (endo=Clusters 6,7,8,14 and15)		22	11	4.5	3.5	
% lacto/endo		32	35	57	40	
% cortico/endo		18	35	7	3	
		AX+ 4days	AX+ 48h00	AXSham	WT	
%cortico/total		4	3.8	0.3	0.3	
%lacto/total		7.2	3.8	2.6	1.4	
% somato/total		1.3	0.7	0.4	0.3	
% gonado/total		7.7	1.8	1.2	1.1	

Endocrine cell cluster numbers from Ax integrated dataset (Fig.5)

Table S3.

Showing 1 to 6 of 6 entries, 5 total columns						
Pairwise for proportion test (Fig.5B)						
	**group1**	**group2**	**p**	**p.adj**	**p.adj.signif**	
**1**	SC Aldh1a2	SC3	5.35E-04	1.02E-02	*	
**2**	SC3	SC Ednrb	1.82E-04	4.18E-03	**	
**3**	SC Aldh1a2	SC4	7.50E-07	2.03E-05	****	
**4**	SC3	SC4	5.55E-13	1.72E-11	****	
**5**	SC Ednrb	SC4	5.76E-04	1.04E-02	*	
**6**	SC Aldh1a2	Prolif	9.91E-05	2.38E-03	**	
**7**	SC3	Prolif	1.50E-06	3.74E-05	****	
**8**	SC Ednrb	Prolif	4.83E-04	1.01E-02	*	
**9**	SC Aldh1a2	Lacto	4.88E-04	1.01E-02	*	
**10**	SC3	Lacto	4.36E-07	1.22E-05	****	
**11**	SC3	Gonado	2.54E-04	5.59E-03	**	
**12**	SC Aldh1a2	Cortico	1.02E-31	3.56E-30	****	
**13**	SC3	Cortico	7.30E-38	2.63E-36	****	
**14**	SC Ednrb	Cortico	1.46E-27	4.97E-26	****	
**15**	SC4	Cortico	1.39E-19	4.60E-18	****	
**16**	Prolif	Cortico	1.36E-06	3.54E-05	****	
**17**	Lacto	Cortico	1.23E-15	3.95E-14	****	
**18**	Gonado	Cortico	6.99E-13	2.10E-11	****	
**19**	Cortico	Somato	3.40E-07	9.87E-06	****	
Showing 1 to 19 of 19 entries, 5 total columns						
Pairwise for proportion test (Fig.6B)						
	n	Statistic	df	p.value	adj.p.value	p.signif
POMC.in.ma	27347	174.098914	1	9.42E-40	2.51E-39	****
PRL.in.males	35836	15.302442	1	9.16E-05	1.47E-04	****
LH.in.males	35836	1.803625	1	1.79E-01	1.79E-01	ns
GH.in.males	31383	10.574053	1	1.15E-03	1.31E-03	**
POMC.in.fem	60958	404.277391	1	6.45E-90	5.16E-89	****
PRL.in.femal	62693	235.560711	1	3.65E-53	1.46E-52	****
LH.in.female	62693	10.638566	1	1.11E-03	1.31E-03	**
GH.in.female	57634	165.802853	1	6.11E-38	1.22E-37	****

Pairwise for proportion test results.

Table S4

SOX2; AQP3 parenchyma manual countings					
	SOX2	SOX2 only	Deduced SOX2;AQP3	% SOX2;AQP3/SOX2	
XY.1	668	228	440	65.86826347	
XY.2	486	210	276	56.79012346	
XY.3	522	128	394	75.4789272	

Fig.2 countings

Sox9;PDGFRb counts were exclusively performed in the parenchyma (cleft excluded) on triple immunofluorescence					
(GFP for Sox9iresGFP, Sox9 to insure nucleus was that of a Sox9 postive cell and PDGFRb)					
Manual coutings					
Animal	Sex	Age	Sox9iresGFP;Sox9	Sox9iresGFP;Sox9;PDGFRb	% Sox9iresGFP;Sox9;PDGFRb/Sox9iresGFP;Sox9
1	XY	6m-old	387	39	10.07751938
2	XY	6 m-old	265	19	7.169811321
3	XY	6 m-old	291	24	8.24742268
4	XY	8 m-old	616	39	6.331168831
1	XX	2 m-old	331	7	2.114803625
2	XX	4 m-old	734	10	1.36239782
3	XX	8 m-old	739	9	1.217861976
4	XX	4 m-old	807	5	0.619578686
Cell countings after lineage tracing using Wnt1Cre on triple immunofluorescence (GFP for Wnt1Cre;R26 eYFP, Sox9 and PDGFRb)					
Manual countings					
Animal	Sex	Age	Sox9;PDGFRb	Sox9;PDGFRb;eYFP	% Sox9;PDGFRb;eYFP/Sox9;eYFP
1	XY	6w-old	49	36	73.46938776
2	XY	6w-old	55	43	78.18181818
3	XY	6w-old	35	26	74.28571429
4	XY	6w-old	33	25	75.75757576
5	XY	6w-old	55	43	78.18181818
6	XY	6w-old	66	62	93.93939394
1	XX	6w-old	21	9	42.85714286
2	XX	6w-old	17	9	52.94117647
3	XX	6w-old	19	11	57.89473684

Fig.3 countings

Dissociated Sox9iresGFP pituitaries were FACSorted and plated 4 days after surgery.						
Immunofluorescent stainings performed as follow: GH and POMC separately, LH and PRL as a double-immuno.						
Automated countings						
Experiment	positive	DAPI	sum	protein	Sex	% of hormone positive cells/DAPI
Ax	20	2617	2637	POMC	Male	0.758437619
Ax	162	5878	6040	POMC	Male	2.682119205
Ax	221	4945	5166	POMC	Male	4.277971351
ShAx	5	3255	3260	POMC	Male	0.153374233
ShAx	25	4183	4208	POMC	Male	0.594106464
ShAx	72	5964	6036	POMC	Male	1.192842942
Ax	33	4224	4257	POMC	Female	0.775193798
Ax	276	10518	10794	POMC	Female	2.556976098
Ax	479	10744	11223	POMC	Female	4.268021028
ShAx	17	6684	6701	POMC	Female	0.253693479
ShAx	251	17558	17809	POMC	Female	1.409399742
ShAx	20	10154	10174	POMC	Female	0.196579516
Ax	9	2836	2845	PRL	Male	0.316344464
Ax	142	7180	7322	PRL	Male	1.93936083
Ax	165	7214	7379	PRL	Male	2.236075349
ShAx	8	3439	3447	PRL	Male	0.232085872
ShAx	119	4631	4750	PRL	Male	2.505263158
ShAx	312	9781	10093	PRL	Male	3.091251362
Ax	48	3331	3379	PRL	Female	1.420538621
Ax	1093	12121	13214	PRL	Female	8.271530195
Ax	415	13231	13646	PRL	Female	3.04118423
ShAx	50	4512	4562	PRL	Female	1.096010522
ShAx	764	17302	18066	PRL	Female	4.228938337
ShAx	83	9743	9826	PRL	Female	0.844697741
Ax	10	2835	2845	LH	Male	0.351493849
Ax	156	7166	7322	LH	Male	2.130565419
Ax	183	7196	7379	LH	Male	2.480010842
ShAx	10	3437	3447	LH	Male	0.29010734
ShAx	110	4640	4750	LH	Male	2.315789474
ShAx	282	9811	10093	LH	Male	2.794015654
Ax	12	3367	3379	LH	Female	0.355134655
Ax	153	13061	13214	LH	Female	1.157862873
Ax	87	13559	13646	LH	Female	0.637549465
ShAx	10	4552	4562	LH	Female	0.219202104
ShAx	130	17936	18066	LH	Female	0.719583748
ShAx	58	9768	9826	LH	Female	0.59027071
Ax	53	2721	2774	GH	Male	1.910598414
Ax	196	6195	6391	GH	Male	3.066812705
Ax	291	7313	7604	GH	Male	3.826933193
ShAx	46	2823	2869	GH	Male	1.603346114
ShAx	178	6409	6587	GH	Male	2.702292394
ShAx	155	5003	5158	GH	Male	3.005040713
Ax	19	2331	2350	GH	Female	0.808510638
Ax	923	11164	12087	GH	Female	7.636303467
Ax	571	9587	10158	GH	Female	5.621185273
ShAx	36	3430	3466	GH	Female	1.038661281
ShAx	1034	19796	20830	GH	Female	4.963994239
ShAx	194	8549	8743	GH	Female	2.218917992

Figure 6B countings

A week after surgeries Sox9iresCreERT2;R26eYFP pituitaries were processed for immunofluorescence on sections

Countings were performed manually on sections

Animal	Age	Sex	Surgery	ACTH;eYFP
1	<=7 w-old	XX	Ax	66
2	<=7 w-old	XX	Ax	34
3	<=7 w-old	XX	Ax	171
4	<=7 w-old	XX	Ax	19
1	<=7 w-old	XY	Ax	84
2	<=7 w-old	XY	Ax	93
3	<=7 w-old	XY	Ax	66
4	<=7 w-old	XY	Ax	47
5	<=7 w-old	XY	Ax	50
6	<=7 w-old	XY	Ax	78

Animal	Age	Sex	Surgery	PRL;eYFP
1	<=7 w-old	XX	Ax	38
2	<=7 w-old	XX	Ax	68
3	<=7 w-old	XX	Ax	44
4	<=7 w-old	XX	Ax	69
5	<=7 w-old	XX	Ax	86
1	<=7 w-old	XY	Ax	1
2	<=7 w-old	XY	Ax	1
3	<=7 w-old	XY	Ax	0
4	<=7 w-old	XY	XY	0

Animal	Age	Sex	Surgery	LH or FSH;eYFP
1	<=7 w-old	XX	Ax	0
2	<=7 w-old	XX	Ax	1
1	<=7 w-old	XY	Ax	0
2	<=7 w-old	XY	Ax	0

Animal	Age	Sex	Surgery	GH;eYFP
1	<=7 w-old	XX	Gx	1
2	<=7 w-old	XX	Gx	1
1	<=7 w-old	XY	Gx	2
2	<=7 w-old	XY	Gx	2

Animal	Age	Sex	Surgery	Prl;eYFP
1	<=7 w-old	XX	Gx	31
2	<=7 w-old	XX	Gx	44
1	<=7 w-old	XY	Gx	1
2	<=7 w-old	XY	Gx	1

Animal	Age	Sex	Surgery	Prl;eYFP
1	<=7 w-old	XX	None	66
2	<=7 w-old	XX	None	73
3	<=7 w-old	XX	None	72

Fig.6C countings

Cre induction efficiency quantification. On the day of dissociation, the central part of the pituitary was kept to quantify the percentage of recombination on cleft-lining SOX9 positive cells.

Manual countings

Animal	Sex	Age	SOX9	SOX9;eYFP	%induction
1	XY	8 w-old	1076	142	13
2	XY	8 w-old	651	100	15.3
3	XY	8 w-old	972	141	14.5
4	XY	8 w-old	1285	223	17.3
1	XX	8 w-old	527	51	9.6
2	XX	8 w-old	990	140	14
3	XX	8 w-old	789	133	16.9
4	XX	8 w-old	1331	204	15.3
5	XX	8 w-old	1260	209	16.6

Figure 6E countings

Automated Prl;eYFP countings in adult dissociated AL Sox9CreERT2;ReYFP 10 days after induction								
Animal	Sex	Age	DAPI	Prl	eYFP	Prl;eYFP		
1	XY	8 w-old	53433	8774	906	0		
2	XY	8 w-old	118487	21203	1507	1		
3	XY	8 w-old	93587	16065	932	1		
4	XY	8 w-old	124853	21223	1373	0		
1	XX	8 w-old	199922	51856	993	46		
2	XX	8 w-old	69807	20337	605	9		
3	XX	8 w-old	91630	38606	2101	23		
4	XX	8 w-old	197278	66190	3898	58		
5	XX	8 w-old	126823	46440	3086	44		
From Fig.6E countings								
%induction		Number of Prl;eYFP			Estimate of Prl cells from SC			
		if 100% induction			(Number of Prl;eYFP/Prl if 100% induction)			
13		0			0			
15.3		6			0.02829788			
14.5		7			0.04357298			
17.3		0			0			
9.6		479			0.92371182			
14		64			0.31469735			
16.9		136			0.35227685			
15.3		379			0.57259405			
16.6		265			0.57062877			
Automated Prl;eYFP countings in adult dissociated AL Sox9CreERT2;ReYFP after induction at P0								
Animal	Sex	Age	DAPI	Prl	eYFP	Prl;eYFP	%Prl;eYFP/eYFP	
1	XY	8 w-old	25808	2986	189	11	5.82010582	
2	XY	8 w-old	20815	2863	131	21	16.0305344	
3	XY	8 w-old	29361	3890	195	8	4.1025641	
4	XY	8 w-old	39251	5309	386	51	13.2124352	
1	XX	8 w-old	10761	1002	66	6	9.09090909	
2	XX	8 w-old	20353	2519	190	5	2.63157895	
3	XX	8 w-old	15532	1914	214	10	4.6728972	
4	XX	8 w-old	24943	3050	169	4	2.36686391	

Fig.6E, F countings

<u>Counting numbers for Fig. 2, 3, 5 and 6.</u>

Table S5.

CELL.SIGNATURE	GENE
Lactotroph	Prl
Lactotroph	Myoc
Lactotroph	Drd2
Lactotroph	Pou1f1
Somatotrophs	Gh
Somatotrophs	Ghrhr
Somatotrophs	Pou1f1
Thryotrophs	Tshb
Thryotrophs	Trhr
Thryotrophs	Dio2
Thryotrophs	Cga
Melanotrophs	Pomc
Melanotrophs	Pax7
Melanotrophs	Tbx19
Melanotrophs	Pcsk2
Corticotrophs	Crhr1
Corticotrophs	Tbx19
Corticotrophs	Pomc
Corticotrophs	Avpr1b
Gonadotrophs	Lhb
Gonadotrophs	Fshb
Gonadotrophs	Gnrhr
Gonadotrophs	Cga
Gonadotrophs	Nr5a1
Gonadotrophs	Foxp2
Endothelium	Cdh5
Endothelium	Angpt2
Endothelium	Pecam
Pericytes	Pdgfrb
Pericytes	Cspg4
Pericytes	Acta2
Macrophages	Cd68
Macrophages	Cd14
Macrophages	Ccr5
Stem cells	Sox2
Stem cells	Sox9
Stem cells	Hes1
Stem cells	Hey1
Stem cells	Fgfr1
Stem cells	Notch1

Cell type signatures.
